# Long-term in vivo imaging of mouse spinal cord through an optically cleared intervertebral window

**DOI:** 10.1038/s41467-022-29496-x

**Published:** 2022-04-12

**Authors:** Wanjie Wu, Sicong He, Junqiang Wu, Congping Chen, Xuesong Li, Kai Liu, Jianan Y. Qu

**Affiliations:** 1 Department of Electronic and Computer Engineering, The Hong Kong University of Science and Technology, Clear Water Bay, Kowloon, Hong Kong, P. R. China; 2 Department of Biology, School of Life Sciences, Southern University of Science and Technology, Shenzhen, China; 3 Division of Life Science, The Hong Kong University of Science and Technology, Clear Water Bay, Kowloon, Hong Kong, P. R. China; 4 State Key Laboratory of Molecular Neuroscience, The Hong Kong University of Science and Technology, Clear Water Bay, Kowloon, Hong Kong, P. R. China; 5 Center of Systems Biology and Human Health, The Hong Kong University of Science and Technology, Clear Water Bay, Kowloon, Hong Kong, P. R. China; 6 Molecular Neuroscience Center, The Hong Kong University of Science and Technology, Clear Water Bay, Kowloon, Hong Kong, P. R. China

**Keywords:** Multiphoton microscopy, Cellular neuroscience, Optical imaging

## Abstract

The spinal cord accounts for the main communication pathway between the brain and the peripheral nervous system. Spinal cord injury is a devastating and largely irreversible neurological trauma, and can result in lifelong disability and paralysis with no available cure. In vivo spinal cord imaging in mouse models without introducing immunological artifacts is critical to understand spinal cord pathology and discover effective treatments. We developed a minimally invasive intervertebral window by retaining the ligamentum flavum to protect the underlying spinal cord. By introducing an optical clearing method, we achieve repeated two-photon fluorescence and stimulated Raman scattering imaging at subcellular resolution with up to 15 imaging sessions over 6–167 days and observe no inflammatory response. Using this optically cleared intervertebral window, we study neuron-glia dynamics following laser axotomy and observe strengthened contact of microglia with the nodes of Ranvier during axonal degeneration. By enabling long-term, repetitive, stable, high-resolution and inflammation-free imaging of mouse spinal cord, our method provides a reliable platform in the research aiming at interpretation of spinal cord physiology and pathology.

## Abstract (web summary)

Wu et al. developed a technique for longitudinal imaging with subcellular resolution of the spinal cord without causing inflammation or microglia activation in live mouse through an optically cleared intervertebral window.

## Introduction

In vivo imaging of the central nervous system (CNS) of small animal models is a crucial means of understanding the function of the CNS and its response to injury or diseases. In recent decades, nonlinear optical (NLO) microscopy has emerged as a powerful tool for the high-resolution imaging of biological tissues, including the CNS. Imaging of the live brain with subcellular resolution has been achieved using NLO microscopy through a cranial window in the mouse skull^1^. In the past decades, a variety of cranial windows for chronic live brain imaging have been developed with various technical refinements for in vivo brain research^2,3^. Specifically, thinned-skull^4,5^ and skull optical clearing protocols^6,7^ have been developed to avoid the inflammatory artifacts caused by surgery. These minimally invasive procedures allow to study cell dynamics under healthy and pathological conditions in the living brain^8–11^. As with the brain, imaging the spinal cord without inflammation induced by surgical preparation has been in high demand for spinal cord studies, including spinal cord injury, multiple sclerosis, neuropathic pain and spinal cord ischemia. However, preparing a spinal window in a mouse is much more challenging than a cranial window because of the more complex gross anatomy and large motion artifacts caused by the heartbeat and breathing.

To obtain optical access to the spinal cord, acute surgical preparation is usually adopted, which requires repetitive surgery for long-term imaging^12,13^. During preparation, the spinal cord is exposed by performing a dorsal laminectomy. Sometimes, dura is removed to increase imaging resolution^12,14^, which inevitably disturbs the spinal cord tissue and increases the risk of surgical trauma. To stabilize the spinal cord during imaging, artificial ventilation, heavy sedation, animal suspension and spine clamping were used to reduce motion artifacts from heartbeat and breathing^12–15^. As a result, high-resolution longitudinal imaging of the spinal cord can be achieved with up to six imaging sessions, limited by the increasing difficulty of repetitive surgery^16–19^. Another method of implanting a spinal chamber can achieve long-term imaging without the requirement of repetitive surgery^20–22^ and allows stable imaging of awake or even freely behaving mice^23^. However, a transient increase in the density of microglia and other inflammatory cells was observed after window implantation, because an immune response was activated in the spinal cord^20,21^. To increase window clarity and tolerance to implants, pharmacologic management of inflammation is required, which may affect the disease process being investigated. Recently, another protocol, spinal cord imaging through the intervertebral spaces without performing a dorsal laminectomy, has been proposed as a less invasive way to provide optical access to the spinal cord^24,25^. By removing muscle and ligament tissues between adjacent vertebrae, the spinal cord was imaged with only dura left. Using this protocol, it is reported that microglia activation was not observed by 2-hour time-lapse imaging after surgery, though clear microglia imaging and quantitative analysis were not demonstrated in the study^24^. Repetitive surgery coupled with an intervertebral window enabled longitudinal imaging with up to ten separate imaging sessions over more than 200 days^25^, which is comparable to the performance of a chronic implanted window^20^.

Despite the less invasive protocol of the intervertebral window, the inflammatory response to this surgical preparation has not been studied well, and it remains unclear whether an intervertebral window can serve as a reliable method to study neuroinflammatory disorders in the spinal cord without surgery-induced artifacts. In this work, we propose an improved intervertebral window protocol, which retains the ligamentum flavum (LF) to significantly decrease the risk of activating microglia. In addition, to overcome the scattering issue induced by the LF and improve the image quality of the spinal cord, we adopted an optical clearing technique using a nontoxic chemical, Iodixanol, to treat the window interface. As an FDA approved contrast agent for coronary angiography^26,27^, Iodixanol has been shown to improve the quality of biological imaging via refractive index matching in live specimens^28^. In this work, Iodixanol improved the spinal cord imaging performance during longitudinal imaging and substantially extended the number of repetitive imaging sessions. Using this optically cleared intervertebral window, we achieved subcellular-resolution, longitudinal imaging of the spinal cord up to 15 imaging sessions over 167 days without an inflammatory activation. With this minimally invasive long-term intervertebral window, we used a multimodal NLO microscope system (Supplementary Fig. 1) to study the neuron-glia dynamics following imaging-guided laser injury of axons. We further investigated the interaction between microglia and the nodes of Ranvier under normal and injured conditions. Different types of dynamic glia-node interaction were classified based on time-lapse imaging, and significantly strengthened contact between microglia and the nodes of Ranvier was observed after the distal axon was injured by laser ablation.

## Results

### Intervertebral window for in vivo imaging of spinal cord

We investigated the behavior of microglia in the spinal cord of Cx3CR1-GFP mice following preparations of a conventional intervertebral window and a new intervertebral window of retaining LF, respectively. The microglial morphology was used as an indicator of inflammatory activity. It is known that microglial cells are the primary immune effector cells in the CNS. In the homeostatic state, microglia are highly ramified and dynamic, with their motile processes continually probing the tissue’s microenvironment^29–31^. On exposure to pathogen- or damage-associated molecular patterns, microglia are activated and change their morphology from ramified to amoeboid with enlarged soma and retracted processes^32–35^. As microglial phenotypes are inextricably associated with their function^36–39^, microglial morphology has been used widely as an objective criterion by which microglia activation and inflammatory activity in the CNS can be identified^38,40–48^. Notably, a number of studies have used a set of morphological parameters to describe the shapes of microglia cells and analyzed their dependence on the level of activation, which was assessed using immunohistochemical staining of cytokine signatures to highlight inflammatory activation^38,41,49–51^. Quantitative analysis showed that the morphology of microglia changes progressively with the expression level of various inflammatory cytokines including IL-1β, IBA-1, CD11b, and CD68^38,41,49,51^. Unlike the immunostaining method that is only applicable to postmortem study, the morphological analysis of microglia combined with high-resolution in vivo imaging techniques can serve as a versatile and sensitive means to detect subtle inflammatory activity in living animals, which is indispensable for in vivo longitudinal study of immune responses to different pathological situations. Among the morphological parameters, the ramification index (RI)^40,52–55^ and the number of process endpoints (NPE)^41,44,56–58^ are widely used to describe the ramification of microglial cells quantitatively. RI is calculated as the ratio of the cell’s perimeter to its area normalized to that of a circle with the same area^40^, while NPE counts the total number of microglial cell processes^41^. Significant decreases in both the ramification index and the endpoints of microglia are typical symptoms of high degrees of inflammatory activation, which is evident in different pathological models of neuroinflammation such as diffuse brain injury, ischemic stroke, peripheral nerve injury, etc^41,52,55,58^. In this study, all the mentioned inflammatory activations in local spinal cord tissue refer to those causing microglial morphological changes.

To study the microglial phenotypes under different conditions, mice were divided into three groups (three mice per group) (Supplementary Fig. 2). The first group of mice had dorsal column crush (DCC) injury performed at the T12 level after a laminectomy to generate an acute inflammatory process, so that the morphology of activated microglia can be characterized first as a positive control. The second group of mice underwent the conventional surgical procedure to expose the spinal cord in the intervertebral gap between the T12-T13 vertebrae. One hour after surgery, mice in these two groups were imaged by a two-photon excited fluorescence (TPEF) microscope for two hours and the behavior of microglia was recorded with a 30-min interval. After imaging, all the mice were perfused for histological analysis. In addition, the third group of mice, which did not undergo any surgery before the histological study, were used as a negative control. Next, we examined the microglial morphology in the fixed spinal cord slices of all three groups using a two-photon microscope (Supplementary Fig. 3a). The microglial cells in the region of 0–50 $\mu$m below the dorsal surface were selected for analysis, corresponding to the in vivo imaging depth. By comparison with the negative control group, the small values of RI and NPE in the positive control group indicates severe activation of microglia after spinal cord injury (Supplementary Fig. 3b,c). Of the three mice with intervertebral windows, one mouse (#2) showed significantly decreased RI and NPE and aggregation of microglia was also found close to the dorsal surface (Supplementary Fig. 3a), while the other two mice showed comparable morphological indices to the negative control group. Then we compared the in vivo results (Supplementary Fig. 3d–f) with those of histopathology studies. The in vivo time-lapse imaging shows that microglia in the spinal cord injured by DCC were activated with significantly decreased ramification at the beginning of imaging and little change over the following two hours (Supplementary Fig. 3e, f). This shows that microglia can respond quickly to pathological insults and be activated within an hour, as previously demonstrated^29^. In the mice with an intervertebral window, microglia showed differentiated but stable ramification during the two-hour observation. Consistently with histological results, the mouse (#2) with activated microglia in the histological analysis also showed activation of microglia with retraction of fine processes in in vivo studies (Supplementary Fig. 3e, f). To rigorously study the probability of microglial activation for the conventional intervertebral window preparation, we performed repetitive imaging for 12 mice and the imaging session for each mouse stopped once microglia activation was observed in the spinal cord. Among 12 mice, 8 mice were imaged only once with 7 mice observed microglial activation at the first imaging session, while one mouse did not show microglial activation but failed to recover from the anesthesia after imaging; 3 mice were imaged twice with microglial activation observed at the second imaging session; Only one mouse went into the third imaging session, but microglia activation was observed in the session. As a result, in a total of 17 imaging sessions performed for 12 mice, only six imaging sessions did not show microglial morphological activation. Therefore, the success rate for the conventional intervertebral window was calculated as 35% (6/17) using a negative binomial distribution statistical model^59,60^. Notably, the difficulty of surgery increased significantly in later procedures due to the growth of scar tissue adhering to the spinal cord surface. This result suggests that conventional intervertebral window preparation will inevitably cause irritation to the spinal cord and induce activation of microglia, preventing the inflammation-free longitudinal study of the spinal cord.

**Fig. 1:**
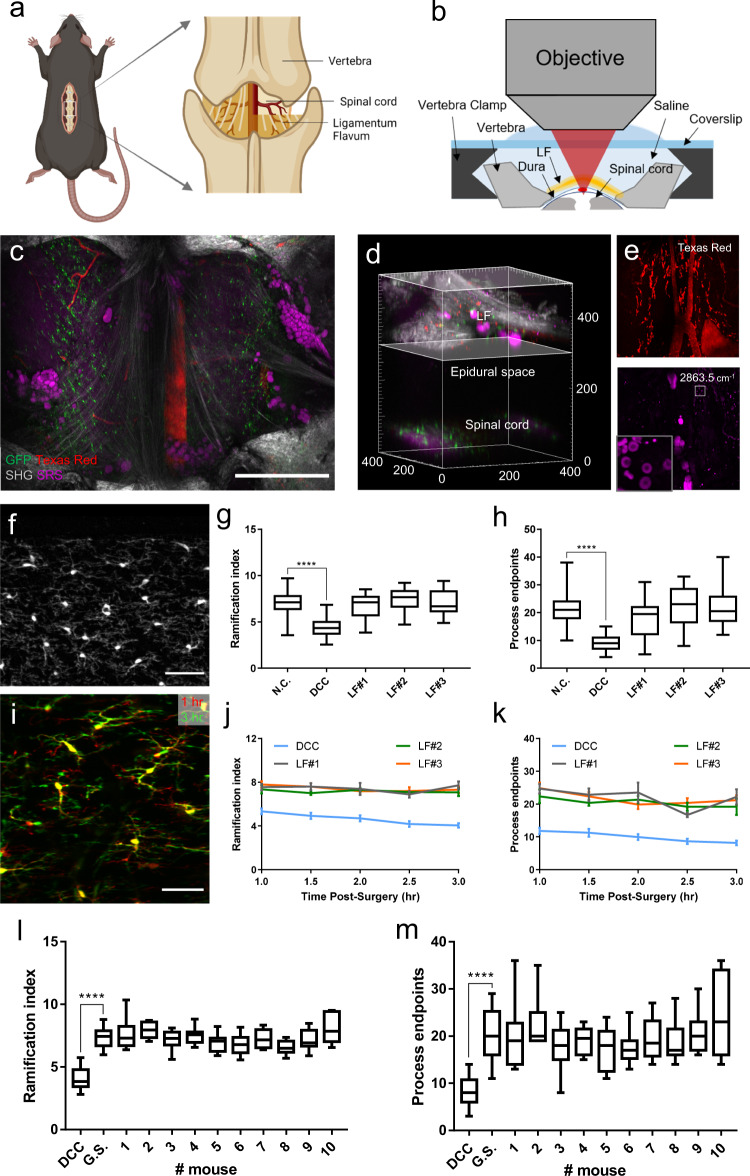
Intervertebral window of retaining ligamentum flavum. **a** Schematic diagram of the intervertebral window with ligamentum flavum (LF). **b** Cross-sectional schematics of the LF window. **c**, **d** Projection and 3D reconstruction of an in vivo multimodal image stack of the LF window. Green: GFP labeled microglia; Red: blood vessels labeled with Texas Red dextran; Gray: second harmonic generation (SHG) signals of collagen and other connective tissues; Magenta: stimulated Raman scattering (SRS) signals of adipose tissue and myelin with the Raman shift at 2863.5 cm^−1^, attributed to the vibration of the methylene group enriched in lipids. Scale bar, 500 $\mu$m. **e** Maximal projection of the image volume between the two planes of depth from 300–500 $\mu$m indicated in (**d**) showing the distribution of blood vessels and adipocytes in the epidural space. Cells labeled by Texas Red in the epidural space are probably invading immune cells. Cells shown in the inset with strong pump-probe absorption at 2863.5 cm^−1^ are red blood cells indicated by their specific dumbbell shape. **f** Two-photon fluorescence image of a 50-$\mu$m-thick longitudinal spinal cord slice under the LF window. Scale bar, 50 $\mu$m. **g**, **h** Evaluation of the microglial ramification index (**g**) and number of process endpoints (**h**) of spinal cord fixed slices from the LF window group, the dorsal column crush (DCC) group and the negative control (N.C.) group. n $\ge$ 20 microglial cells were analyzed from 6–8 slices per mouse, three mice per group. **i** The in vivo superimposed images of microglia at 1 hour and 3 hours post-surgery, showing ramified microglia with highly motile processes under the LF window. Scale bar: 50 $\mu$m. **j**, **k** Changes of the microglia ramification index (**j**) and number of process endpoints (**k**) during two-hour in vivo imaging in the LF window group and the DCC group. Images of the same region were taken at an interval of 30 minutes. 6–8 microglial cells in the same imaged region were analyzed per time point per mouse. Error bars, s.e.m. **l**, **m** In vivo evaluation of the microglial ramification index (**l**) and process endpoints (**m**) of ten mice with LF window at the first live imaging session. The in vivo morphological indices from the three non-activated mice with LF (LF#1–3 in (**j**–**k**)) were used as the gold standard (G.S.) for in vivo microglia activation evaluation. The in vivo results from the DCC group were used as the positive control. Microglia activation in each mice was determined by comparing the calculated ramification index and number of process endpoints with the G.S. 7–12 microglial cells were analyzed for morphological quantification for each mouse; the box plots are shown with median, upper and lower quartiles and max and min values. Kruskal-Wallis test: ****P $<$ 0.0001. Data of the DCC and N.C. groups are shared with Supplementary Fig. 3. Images shown in (**f**) and (**i**) are representative results of three experiments in three mice. Figure (**a**) created using BioRender (https://biorender.com/). Source data are provided as a Source Data file.

Prompted by the thinned-skull procedure, we explored whether we can lower the risk of inflammation by retaining the LF during surgical preparation of the intervertebral window (Supplementary Fig. 4). The LF is a series of ligaments composed of elastic fibers and collagen. They join the laminae of the adjacent vertebra and are located directly above the spinal cord from a posterosuperior view, separated by the meninges and epidural space (Fig. 1a, b)^61,62^. The epidural space contains adipose tissue and blood vessels, which, together with LF, protects the underlying spinal cord, but makes the whole window optically inhomogeneous and less transparent (Fig. 1c–e, Supplementary Fig. 5). A small number of cells labeled by Texas Red Dextran in the epidural space are probably phagocytic immune cells, which is also observed in previous studies^20,21,63^ (Fig. 1e). Nevertheless, we found that high-resolution images of the spinal cord could still be captured in a small field of view (FOV) where there are no adipose tissue and blood vessels along the optical path (Supplementary Fig. 5).

To evaluate the activation of microglia beneath the new intervertebral LF window, we characterized the morphology of microglia both in vivo and in fixed spinal cord using high-resolution TPEF imaging, and compared it to that of intact and injured spinal cords. Histological results show that microglia under the window showed ramified morphology with similar RI and NPE to the negative control group (Fig. 1f–h). Meanwhile, in vivo time-lapse imaging suggests that microglia retained ramified morphology during the two-hour imaging period (Fig. 1i–k). To validate the repeatability of the surgical preparation of the new window, we imaged another ten mice through the LF window and conducted quantitative morphological analysis of microglia. Single imaging session was performed for each mouse and the morphological parameters were compared to the gold standard, the same parameters obtained from the three non-activated mice with LF (LF#1–3 in Fig. 1j, k). Notably, no evident changes in microglial morphology were detected, implying no activation in response to surgical preparation of the LF window, at least after ten independent trials with ten mice (Fig. 1l, m). This result indicates that retaining LF can indeed prevent microglia activation, and thus this protocol of LF window can serve as a minimally invasive method for in vivo optical imaging of the spinal cord.

### Optical clearing intervertebral window of retaining LF

**Fig. 2:**
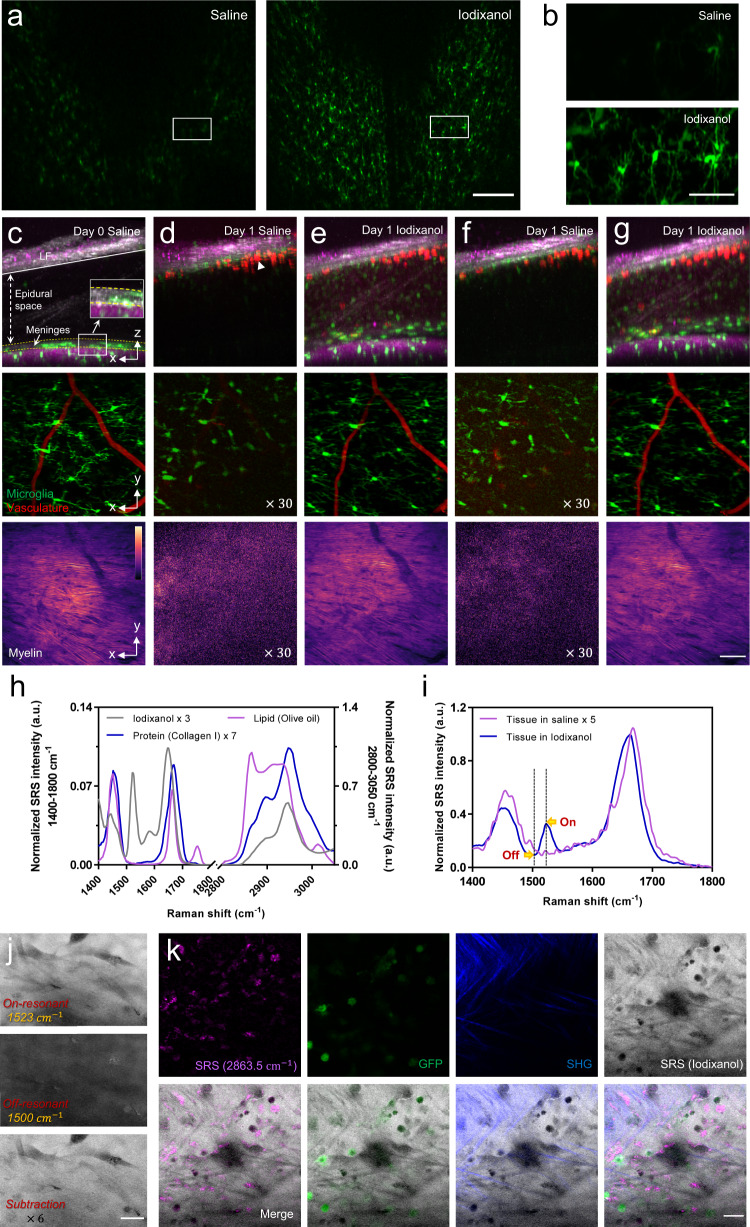
Optical clearance of intervertebral LF window by using Iodixanol. **a** Maximal projection of a microglia TPEF image stack under LF window before and after optical clearing on day 1. Scale bar, 200 $\mu$m. **b** Magnification of the box region in (**a**) shows the detailed microglia image before and after optical clearing. Scale bar, 50 $\mu$m. (**c**–**g**) Top row: Maximal x-z projection of the multimodal images of the LF window. Green, GFP-labeled microglia and other immune cells; Gray: SHG of connective tissues; Red: Texas Red labeled blood vessels and invading immune cells in the epidural space (white arrowhead); Magenta, SRS of lipid at Raman shift of 2863.5 cm^−1^; Middle row: the x-y maximal TPEF projection images of microglia (green) and vasculature (red) under LF window; Bottom row: the x-y maximal SRS projection images of myelin under LF window. The images were captured on day 0 (**c**) and day 1 (**d**–**g**) after first surgery. On day 1, the window was firstly immersed in saline (**d**) and then replaced with Iodixanol (**e**) which was removed (**f**) and reapplied (**g**) again at later times to verify the repeatability of the clearing effect. All the x-z and x-y projection images were normalized to the same value for each imaging modality. The signal intensity in x-y projection images of (**d**) and (**f**) was digitally enhanced by 30 times for better visualization. In x-z image of (**c**), solid line shows the lower boundary of LF, yellow dashed area indicates the location of meninges and the double-headed arrow indicates the area of epidural space. The inset shows the meninges layer of enhanced SHG signal. Scale bar for x, y, z dimensions, 50 $\mu$m. **h** The SRS spectra of Iodixanol (60% w/v), protein (Type I collagen) and lipid (Olive oil) at the fingerprint (1400–1800 cm^−1^) and Carbon-Hydrogen stretching region (2800–3150 cm^−1^). The spectral intensity was subtracted by the non-SRS background with the flat spectral response and was normalized by the lipid CH_2_ peak at 2863.5 cm^−1^. The SRS spectral intensity of Iodixanol and protein were digitally enhanced 3 and 7 times for better visualization. **i** On day 9, SRS spectra of the LF layer immersed in saline and 60% w/v Iodixanol, respectively. The two spectra were normalized by the peak intensity value of the Iodixanol immersed tissue at a vibrational frequency of 1663.3 cm^−1^. The SRS spectral intensity of tissue immersed in saline was digitally enhanced 5 times for better visualization. **j** The SRS image of the LF layer immersed in 60% Iodixanol at 1523 cm^−1^ (on-resonant) and 1500 cm^−1^ (off-resonant) Raman shift and their subtraction. Scale bar, 20 $\mu$m. **k** The in vivo multimodal NLO images of the Iodixanol immersed LF layer on day 2 showing the distribution of Iodixanol in the interstitial space. Scale bar, 20 $\mu$m. A.u., arbitrary units. Images shown in (**a**) and (**b**) are representative results of six experiments in six mice. Source data are provided as a Source Data file.

Although retaining the LF helps to reduce the risk of inflammation caused by surgery, the LF layer as well as tissues in the epidural space introduces optical scattering and results in decreased penetration depth of spinal cord imaging. After the initial surgery, both LF and the epidural space were infiltrated and filled by a large number of cells, which greatly decreased image contrast and resolution (Supplementary Fig. 6). In this work, an optical clearing method was developed to reduce the optical inhomogeneity of the LF window. We tested the applicability of Iodixanol as an optical clearing medium to facilitate in vivo spinal cord imaging through the LF window. On day 1 postsurgery when cell infiltration largely reduced the transparency of the window, we applied Iodixanol on top of the LF layer and found a significant improvement in the window transparency and optical homogeneity after 10 min (Fig. 2a, b). The application of Iodixanol restored the image contrast and resolution of both two-photon and stimulated Raman scattering (SRS) imaging on day 1 to almost the same level as on day 0 (Fig. 2c–e). Importantly, this improvement can be lost by replacing the Iodixanol with saline (Fig. 2f) and then recovered by reapplying Iodixanol (Fig. 2g). This phenomenon indicates that the reduction in optical inhomogeneity should be achieved by refractive index matching rather than by direct removal of scatterers in tissues. Multimodal imaging of the spinal cord with epidural space and LF showed the tissue structure to be consistent before and after Iodixanol application (Fig. 2e–g), which further confirms our hypothesis concerning the optical clearing mechanism of Iodixanol. By increasing the concentration of Iodixanol up to 60% w/v (*n ≈1.429*), the improvement in imaging increased further, indicating better matching of refractive indices (Supplementary Fig. 7).

Next, we investigated how refractive index matching was achieved in the LF window by using hyperspectral SRS imaging combined with two-photon microscopy. We first acquired the SRS spectrum of Iodixanol in the fingerprint region (1400–1800 cm^−1^) as well as in the Carbon-Hydrogen (C-H) stretching region (2800–3150 cm^−1^) and compared it with typical spectra of lipid (olive oil) and protein (type I collagen) (Fig. 2h). Iodixanol shows a similar spectrum to protein from 2800 to 3150 cm^−1^, but has a quite different spectrum profile in the fingerprint region where there is a unique vibrational peak at 1523 cm^−1^ contributed by the aromatic ring as well as the secondary amide II band in its molecular structure^64^. By sweeping the SRS spectrum of the LF layer immersed in Iodixanol, we found a small SRS peak at 1523 cm^−1^ that disappeared when Iodixanol was rinsed out (Fig. 2i). Therefore, 1523 cm^−1^ is a vibrational peak contributed solely by Iodxianol, which can be used to visualize the Iodixanol distribution without interference from other endogenous biomolecules. To eliminate non-Raman background interference, a subtraction method was used to obtain the genuine SRS signal of Iodxianol (*I*_SRS_ = *I*_ON_ - *I*_OFF_)^65,66^. Briefly, the SRS baseline signal (off-resonant, *I*_OFF_) at 1500 cm^−1^ was subtracted from the SRS peak signal (on-resonant, $I$_ON_) at 1523 cm^−1^ to suppress non-Raman backgrounds (Fig. 2j). To investigate the distribution of Iodixanol through the LF window, we applied multimodal NLO microscopy combing SRS, TPEF and second harmonic generation (SHG) to visualize Iodixanol, cells and collagens simultaneously. The multimodal images showed that collagen and cellular structures are spatially correlated with the negative contrast regions in the Iodixanol SRS images (*I*_SRS_) (Fig. 2k), indicating that Iodixanol achieved refractive index matching primarily by increasing the refractive index of the interstitial fluid. In addition, we didn’t observe any Iodixanol SRS signal inside the spinal cord tissue, which indicates the absence of massive Iodixanol penetration through the meninges. The clearing effect of Iodixanol becomes worse with time due to the gradual dilution of the Iodixanol indicated by the decreased SRS signal of Iodixanol between the window surface and coverslip (Supplementary Fig. 8, Supplementary Video 1). Therefore, Iodixanol was supplemented hourly to maintain good refractive index matching, and the imaging was usually started 10 min after every Iodixanol administration when its optical clearing effect reached a plateau (Supplementary Fig. 8). In addition, optical clearing is equally efficient in both the cervical and lumbar regions in comparison with the T12-T13 lower thoracic region, with the transparency and homogeneity of the LF window significantly improved within 10 mins after Iodixanol administration (Supplementary Figs. 9 and 10). Stable in vivo spinal cord imaging through the optically cleared LF window can also be achieved in the cervical and lumbar regions with similar mouse stabilization scheme (Supplementary Figs. 9 and 10, Supplementary Videos 2 and 3).

Safety issues are a crucial concern when applying optical clearing agent to living animals. Because of nontoxicity, Iodixanol has long been used as an intravenous X-ray contrast agent^26,27^ as well as a density gradient medium for cell isolation^67^. When applied as a refractive index matching media for live imaging, Iodixanol doesn’t show any toxic effects on living Hela cells, planarians and zebrafish^28^. Since microglia can sense subtle changes in their microenvironment through a variety of surface receptors and quickly respond to the changes with their morphology changed^29,68^, in this study, we assessed the effects of exposing the spinal cord to Iodixanol by exploring microglia activation after optical clearing. We imaged microglia through the LF window before and after applying Iodixanol (60% w/v) on day 0 when high-resolution microglia images still could be acquired without optical clearing. Images were taken at 1 h after Iodixanol application for toxicity analysis, allowing microglia to fully react to Iodixanol with potential morphological alterations^31,35^. Results show that microglia remained ramified and continually surveying the microenvironment with highly motile processes^40,42,43^ after Iodixanol administration (Supplementary Fig. 11, Supplementary Video 4). To further assess the potential long-term effects of exposing the spinal cord to Iodixanol, we continued to image microglia on day 1 and day 3 with the window treated with Iodixanol (60% w/v). Time-lapse in vivo imaging shows that all the microglial cells in the FOV retained ramified morphology with dynamic processes, indicating no inflammation (Supplementary Fig. 11, Supplementary Video 4). Collectively, these results demonstrate that applying Iodixanol to the intervertebral window does not impact the spinal cord, largely alleviating safety concerns. We also tested widely reported optical clearing agents^69–72^, glycerol (Supplementary Fig. 12) and PEG400 (Supplementary Fig. 13). We found that both agents induced activation of microglia and offered limited improvement for two-photon imaging through the window.

**Fig. 3:**
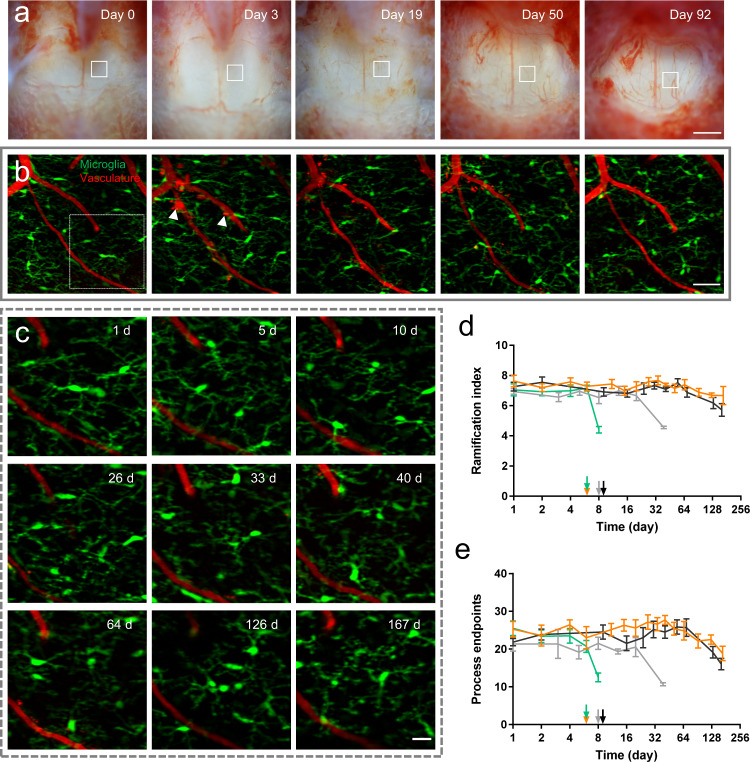
Long-term spinal cord imaging through optically cleared LF windows. **a** Bright-field images of an LF window over three months. Scale bar, 500 $\mu$m. **b** Maximal projections of microglia (green) and vasculature (red) image stacks at the same site in the box region of (**a**). Vasculature labeled by Texas Red dextran was used to navigate to the same region of interest in different imaging sessions. The arrowheads indicate Texas Red dextran labeled perivascular cells and immune cells above the spinal cord. Scale bar, 50 $\mu$m. (**c**) Magnification of the box region in (**b**) shows the detailed structures of microglia and vasculature at indicated times. Scale bar, 50 $\mu$m. (**d**, **e**) Ramification index (**d**) and number of process endpoints (**e**) as functions of time during longitudinal two-photon fluorescence imaging. Each curve represents the statistical data of one mouse. Arrows indicate timing of the first surgical clearing of the overgrowing tissue for each mouse. Surgical clearing of overgrowing tissue was performed thereafter for every imaging session. For statistics of ramification index and number of process endpoints, 6–10 microglial cells were analyzed for each mouse at each time point. The longitudinal study was terminated when microglial activation was observed, or the imaging region of interest was lost because of the shrunken field of view. Error bars, s.e.m. Source data are provided as a Source Data file.

Since optical clearing by Iodixanol can significantly increase the window clarity without activating microglia, we next explored the potential of this optically cleared intervertebral window for minimally invasive longitudinal imaging. We conducted repetitive multimodal NLO imaging on four Cx3CR1-GFP mice and the longitudinal imaging session was terminated once microglial activation was observed (Fig. 3a–c). The results show that longitudinal imaging of more than 10 imaging sessions over 4–5 months without activating microglia was achieved on two of the four mice (Fig. 3d, e). It was found that on day 0, optical clearing significantly increased the transparency and optical homogeneity of the whole window (Supplementary Fig. 14). Within the first week after initial surgery, scar tissue at the surgical site has not developed fully and the large interstitial space below the LF layer allowed easy matching of refractive indices by Iodixanol and therefore permitted high-resolution fluorescence imaging **(**Supplementary Fig. 15**)**. The improvement of fluorescence and SRS signal by optical clearing on day 4 reached about 20 times **(**Supplementary Fig. 15d–f**)**. Usually a week later, scar tissue developing with collagen, blood vessels, and recruited dense cells, severely degraded the window transparency and reduced the optical clearing effect (Supplementary Figs. 15 and 16). Therefore, it is necessary to remove the newly grown tissue above the LF layer. Due to the mechanical toughness of the LF layer, the loose granulation tissue at the top of the window can be easily distinguished. The precise surgical removal of scar tissue leaving the LF layer intact can be achieved with a high success rate. After tissue removal and Iodixanol treatment, spinal cord images with subcellular resolution could be recovered (Supplementary Fig. 16). During each imaging session, to equilibrate the heterogeneous refractive indices, Iodixanol was applied to the surface of the intervertebral window prior to NLO imaging. Although the structure of the LF window varies with time, optimal refractive index matching was always reached at about 10 min after Iodixanol treatment (Supplementary Fig. 15g). Therefore, Iodixanol was supplemented every hour and imaging was usually started 10 min after every Iodixanol administration. It is worth pointing out that although invading cells labeled by Texas Red dextran were observed in the epidural space at the surgery site (Supplementary Fig. 15a), obvious increase of macrophage density in the meninges^73^ was not observed during longitudinal spinal cord imaging (Supplementary Fig. 15c).

We evaluated the inflammation response by morphological analysis of microglia in each imaging session. Microglial cells in the same region of interest (ROI) with all microglial processes appearing in the FOV were selected for morphological quantification. Since the image quality of a microglia is determined by its fluorescent protein expression level and excitation conditions, microglia of low image quality (cell body contrast < 0.97) were excluded for morphological analysis to avoid underestimation of microglial ramification (Supplementary Fig. 17). The statistics of RI and NPE show that microglial activation with significantly decreased ramification was observed on day 8, day 39 and day 161 in three mice (Fig. 3d, e), respectively, due to accidental touch to the spinal cord by surgery tools during the removal of scar tissues. One mouse of four was imaged for as long as 167 days without inflammation (Fig. 3a–c), but the ROI was lost on day 203 because of the decreased FOV of the intervertebral window (Supplementary Fig. 18). From the bright-field images of the two mice which were imaged for more than 160 days, we observed the intervertebral window becoming smaller over time because the newly grown tissue attached to vertebrae became more difficult to remove, probably resulting from fibrotic scarring or damage to the cartilage (Supplementary Fig. 18), which limits the time span of the intervertebral window. To extend the FOV, spinal cord imaging through the adjacent two intervertebral windows can be performed (Supplementary Fig. 19). For the two-photon and SRS microscopy, the imaging depth in the spinal cord dorsal column is about 50$\,{{{{{\rm{\mu }}}}}}{{{{{\rm{m}}}}}}$ below the pia mater (Fig. 2c, e, Supplementary Fig. 5, Supplementary Videos 2 and 3), or 400 ${{{{{\rm{\mu }}}}}}$m below the LF layer (Fig. 1d, Supplementary Fig. 5). This penetration depth is mainly limited by the strong scattering of the lipid-enriched myelin sheaths^74,75^.

### Multimodal NLO imaging of axonal degeneration after laser axotomy

**Fig. 4:**
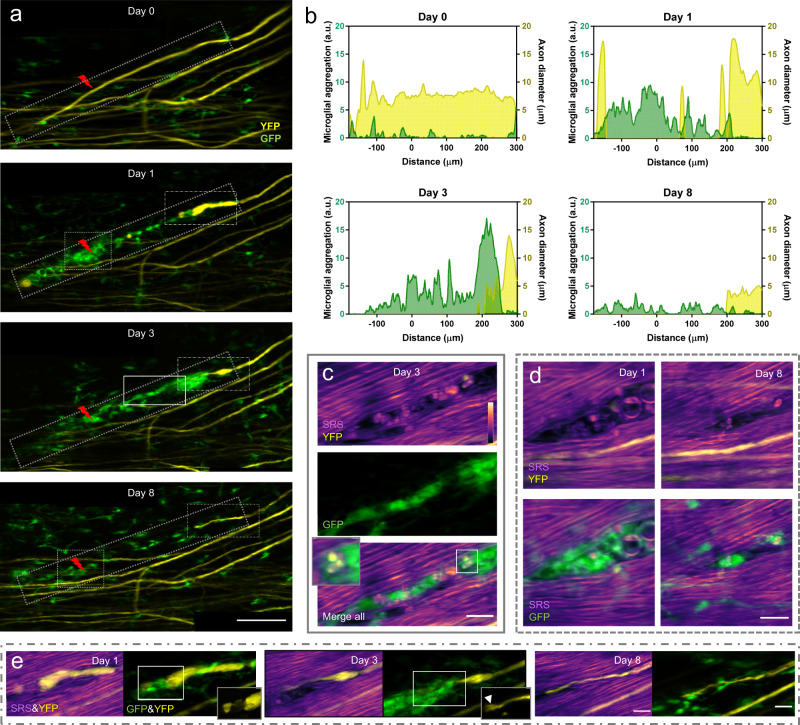
In vivo multimodal NLO imaging of axonal degeneration after laser axotomy. **a** Maximal z intensity projections of TPEF image stacks of YFP labeled axons (yellow) and GFP labeled microglia (green) at indicated times before and after laser axotomy. The lightning bolt symbol indicates the lesion site. Scale bar, 100 $\mu$m. **b** The dynamics of the distribution of microglia (green) along the axonal degeneration path and the diameter of the injured axon (yellow). Only microglial cells located in the dot rectangular region along the degenerating axon in (**a**) were included for analysis. **c** The multimodal image of the spinal cord in the solid box region in (**a**) shows resident microglia and/or bone marrow-derived macrophage (BMDM) aggregation along the axonal degeneration path at 3 days postinjury (dpi). Colocalization of axon (yellow) and myelin (magenta) debris and microglia (green) indicates phagocytosis of microglia/BMDMs. Insets, a zoomed-in view of myelin and axon debris colocalized with microglia/BMDMs. **d** The multimodal images taken at 1dpi and 8 dpi in the dashed box region in (**a**) show the initialization and finalization of debris clearance, respectively. **e** The zoomed-in multimodal images of the axonal proximal end at indicated time points. The imaging area corresponds to the long dash-dot box region in (**a**). For clear visualization, the merged SRS and YFP images are shown as a single slice, while the merged GFP and YFP images are shown as the maximum z projections of image stacks. Insets, YFP images of the axonal proximal end. The arrowhead denotes small sprouts emerging from the tip of the axon. SRS images of myelin were taken at Raman shift of 2863.5 cm^−1^. Scale bars in (**c**–**e**), 20 $\mu$m. A.u., arbitrary units. Source data are provided as a Source Data file.

Using double transgenic mice expressing EYFP in dorsal root ganglion afferent neurons and EGFP in microglia, we evaluated the response of axons and microglia to laser-induced injury. Axons and surrounding myelin sheaths were imaged together with microglia using our multimodal NLO microscope. YFP and GFP signals were differentiated using a spectral unmixing method as previously described^76^. We conducted precise single axon axotomy using tightly focused femtosecond laser pulses (Fig. 4, Supplementary Fig. 20). Microglia responded rapidly to the injury by extending their cytoplasmic processes towards the lesion^29,30^ (Supplementary Fig. 20b, Supplementary Video 5). The proximal end of the axon underwent acute degeneration within an hour post injury^14,18^, and the surrounding myelin sheath kept close contact with the axon during degeneration (Supplementary Fig. 20c). After 1 day, dieback of the proximal ends slowed down and a large number of microglia, as well as bone marrow-derived macrophages (BMDMs) were recruited to the injury site^77^. Thanks to the precise laser axotomy on a single axon and high-resolution in vivo fluorescence imaging^18,19,78–80^, we could observe clearly the spatially confined microglia/BMDM distribution strictly along the axonal degeneration path (Fig. 4a). Though the influx of microglia and BMDMs has been shown to correlate with axonal dieback^77,81^, the imaging-guided laser microsurgery along with longitudinal imaging permits the study of the interaction between microglia/BMDMs and injured axons in a much higher resolution both temporally and spatially. The results show that 1 day postinjury (dpi), microglia/BMDMs mainly aggregated at the lesion site. At 3 dpi, however, the microglia/BMDM aggregation moved along the direction of axon degeneration and kept physical contact with the proximal end of the injured axon. At 8 dpi, the aggregation disappeared and microglia/BMDMs were redistributed homogeneously in the FOV (Fig. 4a, b). This spatiotemporal distribution of microglia/BMDMs could be correlated with its cellular function of tissue debris clearance. At 3 dpi, microglia/BMDMs phagocytosis of the myelin and axon debris along the axonal degeneration path was observed (Fig. 4c). Our time-lapse multimodal imaging showed that the amount of myelin debris was significantly reduced, corresponding with the decreased density of microglia/BMDMs at 8 dpi, with only a few pieces of debris left inside the cell bodies of myelin-laden microglia/BMDMs (Fig. 4d). These results provide crucial in vivo evidence to support previous studies that observed microglia/BMDM engulfment of axon and myelin debris based on postmortem analysis^82,83^.

In addition to the phagocytosis of axon and myelin debris, microglia and BMDMs were also reported to mediate axonal dieback by forming cell to cell contacts with the dystrophic endings of injured axons^77,81,84^. At 1 dpi, the injured axon formed an enlarged endbulb where the surrounding myelin sheath was lost (Fig. 4e). Interestingly, an axonal fragment was loosely connected to the enlarged proximal ends and surrounded by microglia/BMDMs (Fig. 4e). On the third day after injury, the axonal proximal end was surrounded by a larger amount of microglia/BMDMs. Despite closely contacted by microglia/BMDMs, the injured axon had limited secondary degeneration after day 1, and conversely, it showed signs of regeneration, indicated by the thinning dystrophic proximal ends and growth cone-like structures at day 3 and day 8. Specifically, the injured axon exhibited a regeneration length of 12 *μ*m from 3 to 8 dpi, as measured from the time-lapse fluorescence images. (Fig. 4e).

In addition, we also investigated microglial behavior and microglia-axon interaction following a more severe spinal cord damage by inflicting laser injury over a large area (Supplementary Fig. 21). Large microglia/BMDM aggregations were observed at the lesion site at 1 dpi and expanded further in the following days. Compared with the phenomena observed in the single axon injury, the strict spatial correlation between the microglia/BMDM distribution and individual axonal degeneration path was not observed, probably because of the large size of the injury. In addition, the microglia/BMDM aggregation remained at the lesion site for at least a month, suggesting long-lasting inflammatory activity. The injured axon underwent acute and subacute degeneration within the first three days and then became almost immobilized in the following weeks. As we observed previously, the axonal ends first became enlarged and then thinned with sprouts appearing at 3 and 8 dpi. As can be seen, by taking advantage of the multimodal NLO imaging with high spatiotemporal resolution, we demonstrated a reliable model to study the highly dynamic processes of debris clearance and glia-neuron interactions during tissue injury and remodeling under finely controlled injury conditions.

### Dynamic interaction between microglia and the nodes of Ranvier

**Fig. 5:**
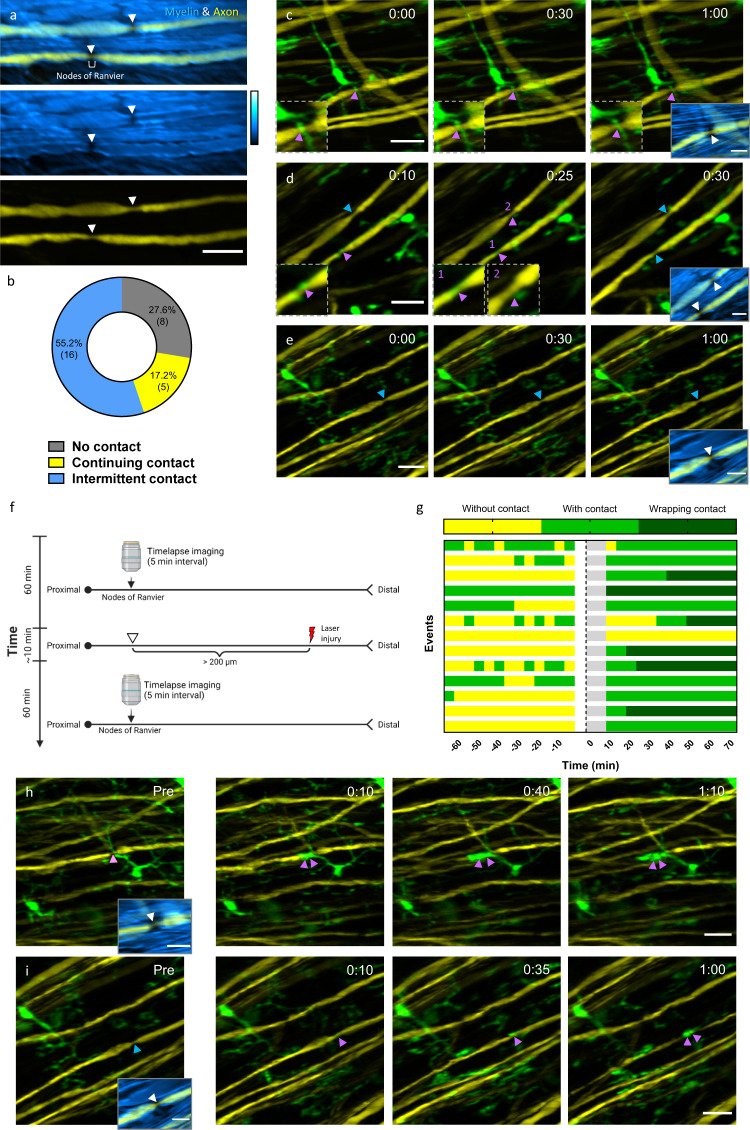
Dynamic contact between microglia and the nodes of Ranvier. **a** The overlay of the SRS image of myelin (blue) and the TPEF image of YFP axon (yellow) shows the structure of the nodes of Ranvier. Arrowheads denote the locations of nodes. Scale bar, 20 $\mu$m. **b** The categorization and statistics of microglial contact with the nodes of Ranvier. Totally 29 nodes from 4 mice were studied. (**c**–**e**) Representative results of continuing contact (**c**), intermittent contact (**d**) and no contact (**e**) between microglia (green) and the nodes of Ranvier (arrowheads) during 1-hour time-lapse imaging. Insets in the dashed box: zoomed-in fluorescence images to clearly show the microglia-node contacts. Insets in the solid box, overlay of the SRS image of myelin (blue) and the TPEF image of axons (yellow) showing the position of nodes (white arrowheads). Purple arrowheads indicate nodes with microglial contact, while blue arrowheads indicate nodes with no microglial contact. Scale bar, 20 $\mu$m. (**f**) Illustration of the experimental design. Single-axon laser axotomy was conducted at more than 200 $\mu$m away from the target node of Ranvier to avoid direct laser-induced microglial activation near the nodes. (**g**) Quantification of microglia-nodes contact before and after laser axotomy. Each bar represents a node of Ranvier. Totally 13 nodes from 4 mice were recorded. The blank and grey areas on the bars denote the time for laser injury and imaging setup when imaging was not performed. Laser injury was performed at time 0. (**h**, **i**) Representative time sequence images of nodes with wrapped contacts with microglia after axon injury. Before injury, the node in (**h**) has continuing contact with microglia, while the node in (**i**) has no contact with microglia. After injury, wrapping contacts with nodes enveloped by microglia processes are indicated with double purple arrowheads. Time post injury is presented as hr: min. Scale bar, 20 $\mu$m. Insets, overlay of myelin SRS image and axon fluorescence image. Scale bar for all the insets, 10 $\mu$m. Source data are provided as a Source Data file.

Nodes of Ranvier, known as myelin-sheath gaps, are characterized by short and periodic regions of the axonal membrane that are bare of myelin^85,86^. The axolemma at nodes of Ranvier is exposed directly to the extracellular matrix and is highly enriched in ion channels, which permit the rapid exchange of ions to regenerate the action potential^86^. Therefore, nodes of Ranvier play a key role in fast saltatory propagation of action potentials. In the CNS, myelinating oligodendrocytes don’t form nodal microvilli, allowing glial cells to contact the uninsulated axolemma directly at the nodes of Ranvier. Using immunofluorescent staining and electron microscopy, a recent study revealed direct contact between microglia processes and the nodes in rat corpus callosum^87^, although the physiological role of the contact remains elusive. Here, we assessed microglia-axon contacts at the nodes of Ranvier in vivo using Thy1-YFP/Cx3Cr1-GFP double transgenic mice and studied the dynamic behavior of microglia-node interactions during axonal degeneration induced by laser axotomy. Specifically, the position of nodes was first confirmed by merging the SRS image of myelin and the TPEF image of YFP-labeled axons. As can be seen, at the nodes of Ranvier the axon is not wrapped by myelin and exhibits a decreased diameter compared with the internode regions (Fig. 5a). Microglial contact with the node was then confirmed by colocalization of the GFP and YFP signals. However, it should be emphasized that the direct contact between microglial processes and nodes of Ranvier cannot be definitely confirmed due to the inherent resolution limitation of the optical microscopy. First, we conducted 1-hour time-lapse multimodal imaging of the spinal cord through the LF window. As expected, the microglial cells under the window displayed ramified morphology with highly motile processes. Interestingly, we observed that a large proportion of nodes (72.4%, *n* = 21) were contacted by microglial processes (Fig. 5b). Notably, there were a small number of nodes (17.2%, *n* = 5) showing continuing contact with microglia during 1-h imaging time, with one of the microglial processes sticking to the node of Ranvier and remaining stable over time (Fig. 5c). Nevertheless, most of the microglia-node contacts were intermittent (55.2%, *n* = 16), occurring as microglial processes randomly scanning over the surrounding environment (Fig. 5d). In addition, we also observed that a microglial cell can access two nodes of Ranvier simultaneously with its highly branched processes (Fig. 5d), showing the diversity of microglia-node interactions. A small portion of nodes showed no contact with the microglia during 1-h time-lapse imaging (Fig. 5b, e). Then we explored this contact at the nodes of injured axons. Time-lapse imaging was performed for an hour before and almost immediately after laser axotomy on a single axon (Fig. 5f). To avoid directly influencing the behavior of microglia around nodes of Ranvier, laser axotomy was performed at least 200 $\mu$m distal to the target node (Supplementary Fig. 22). After every laser injury, we monitored the dynamics of the microglia around the node and found that their processes were not recruited to the lesion site, suggesting that this precisely controlled distal injury method can exclude the laser-induced microglial response and thus provides an ideal means to study the specific microglial behaviors related to axonal degeneration.

Strikingly, we found that after injury at the distal end of the axon, the nodes of Ranvier were contacted by microglial processes significantly more frequently (Fig. 5g). Among the 12 nodes of Ranvier which had no contact or intermittent contact with microglia before laser injury, 10 nodes attracted microglial processes within 15 min after axotomy and remained in continuing contact during the following hour. Moreover, in some cases, microglial processes were intensively recruited and fused to the nodes, forming an enlarged containment around the nodes (Fig. 5h, i, Supplementary Videos 6–8). This wrapping contact was observed in about half the nodes (6/13). These results show that the microglia-node contact is strongly regulated in the injured axons. Although the mechanisms underlying the pronounced changes in microglia-node interactions remain unclear, it is suggested that microglia dynamics can be modulated by the concentration of cations such as potassium (${K}^{+}$)^40^ and calcium (${{Ca}}^{2+}$)^88,89^. At the nodes of Ranvier, where action potentials regenerate, large amount of ${K}^{+}$ and ${{{{{{\rm{Na}}}}}}}^{+}$ are rapidly exchanged on the uninsulated axolemma^86^. In addition, calcium influx through the nodes is reported to happen in a manner dependent on neuron activity^90,91^. Therefore, microglia contact with nodes of Ranvier may be closely associated with the change of ${K}^{+}$ and/or ${{Ca}}^{2+}$ concentrations around the nodes. As laser axotomy disrupts the axolemma and surrounding myelin, it would cause a rapid depolarization and an occasional burst of action potentials^92^. Therefore, it is rational to speculate that laser axotomy may affect the concentrations of cations around the node, which further modulates microglia-node contact. Meanwhile, as a unique pathological response triggered by axon injury, the strengthened contact between microglial processes and nodes of Ranvier may offer valuable insights into the regulation of axon-glia interactions during the neurodegeneration process. With this in vivo spinal cord imaging method based on the minimal-invasive intervertebral window and multimodal NLO microscopy, we demonstrated a promising way to study the dynamic interaction between the nodes of Ranvier and microglia under normal and injury condition, opening a door for future studies associated with the functions of the nodes of Ranvier.

## Discussion

Since spinal microglia plays a crucial regulatory role in homeostasis^29^, neurodevelopment^93,94^, and neuronal degeneration or regeneration^77,94–96^, during the surgical preparation of the intervertebral window for chronic imaging, it is of great importance to avoid activating the spinal microglia in order to maintain the native microenvironment of the spinal cord. In this study, we demonstrated the use of a minimally invasive intervertebral window with an optical clearing method to achieve long-term (6–167 days), repetitive (4–15 times), high-resolution (subcellular structure-resolved), and most importantly, inflammation-free (microglia inactive) imaging of mouse spinal cord in vivo. To improve the integrity and rigidity of the intervertebral window, we retained the LF to serve as a buffer for any mechanical force to the spinal cord caused by surgery. This is a key procedure to protect the underlying spinal cord tissue and dramatically reduce the possibility of window preparation activating inflammation. A side effect is that newly generated tissues above and below the LF will gradually lower the window’s clarity and reduce the quality of imaging. To solve this problem, we gently removed the newly grown tissues above the LF, and more crucially, we applied an optical clearing method using Iodixanol as the clearing medium to reduce the photon scattering caused by the window and successfully restore subcellular imaging resolution for more than 160 days. Importantly, by monitoring the morphology of microglia after optical clearing using high-resolution two-photon imaging, we confirmed that administering Iodixanol on the surface of the window does not activate an inflammatory response in the spinal cord, making it a reliable way to improve imaging performance without disturbing spinal homeostasis. Compared with the prior laminectomy and intervertebral windows^16,18,25^, the Iodixanol-based optical clearing technique allows us to remove less tissue above the spinal cord without compromising imaging quality, thus reducing the risk of activating inflammation during the surgical preparation and significantly extended the number of imaging sessions. Based on the experimental results from four mice (Fig. 3d, e), among the totally 38 (5+7+11+15) imaging sessions, microglial activations were observed in the last sessions of three mice, while no microglial activation was observed in the fourth mouse during all the 15 imaging sessions over 167 days. Assuming the activation of microglia for the fourth mouse was observed in 16th imaging session, the minimal success rate was 90% (35/39) according to the statistical analysis based on the negative binomial distribution^59,60^. As a result, this optically cleared LF window of over 90% success rate offers a reliable tool for the longitudinal study of chronic disorders in the spinal cord, such as multiple sclerosis^97,98^, spinal cord injury^99^ and neuropathic pain^100^. It is also worth pointing out that all the surgical procedures require precision and good surgical skills. Thus, proper training is necessary.

The microglial morphology was used as an in vivo indicator of inflammatory activity. It has been reported previously that microglial morphological changes would not always be the earliest sign in response to inflammation^49,50^. In addition, as well as the two conventional forms of resting and activated state, microglia may display an intermediate state in which cells preserve a branched morphology under pathological stimuli^38^. This is because microglial activation and the resultant morphological transformation is a gradual process, and may have diverse responses to pathological conditions and functional states^39,101^. Indeed, this progressive, heterogeneous alteration in microglial morphology during the activation process may disturb the accuracy of judgments of the activation states of individual microglia based on morphology. Nevertheless, it is widely accepted that inflammation in local tissues can be determined objectively by rigorous statistical analysis of the average morphological parameters of a large population of microglia in the ROI^38,44,55^. Furthermore, it has been observed that microglial activation in response to acute CNS injury is usually rapid and most of the microglial cells near the lesion site can quickly retract processes and even acquire the amoeboid phenotype within a few hours of the stimulus^30,102^. Therefore, in order to assess the extent of inflammatory activation during the preparation of a spinal cord window, we conducted quantitative and statistical characterization of the ramification index and process endpoints of the spinal cord microglia using time-lapse in vivo imaging.

By using a home-built multimodal NLO microscope system that integrates TPEF, SHG and SRS imaging, we achieved simultaneous visualization of a variety of structures in and above the spinal cord, including axons, myelin, microglia, blood vessels, collagen, lipid, etc., facilitating our understanding of the remodeling of the complex microenvironment in the intervertebral window during longitudinal imaging. This multimodal imaging plays a crucial role in characterizing the biophysical and biochemical properties of the intervertebral window, monitoring the axon-glia dynamics following laser injury, and identifying the microglial contacts with the nodes of Ranvier. In addition, we adopted two-photon laser microsurgery^78–80^ to study spinal cord injury in a well-controlled manner, which can achieve precise injury of single axon in the dorsal column area^18,19^. As well as the advanced imaging tool, another indispensable factor for high-resolution spinal cord imaging is that we established a custom-designed stabilization stage to minimize the influence of mice breathing during imaging, and also applied rigorous image registration algorithms to correct residual motion artifacts. Combined with a redesigned mechanical stabilization device^23^ and a miniaturized two-photon microscope system^103^, this optically cleared LF window may be used for imaging awake or even freely behaving mice.

It should be noted that although the sub-cellular resolution of two-photon spinal cord imaging can be achieved most of the time through tissue removal and optical clearing, clear images may be hard to acquire when newly generated blood vessels in the epidural space are densely distributed right above the ROI. Further, optical clearing showed smaller improvement for SRS imaging compared to two-photon imaging. This probably results from the chromatic aberration introduced by the optically cleared LF window since SRS generation depends critically on the spatial overlap of the pump and Stokes beams at the focal point. To further improve the image quality under the LF window in the future, adaptive optics could be introduced and integrated into the NLO microscope to compensate for the monochromatic and chromatic aberrations caused by the window. It is also worth noting that the effective area of the intervertebral window decreased significantly after 3 months because of the growth of surrounding tissues that are difficult to remove. Therefore, to avoid losing the longitudinal traced ROI due to the decreased FOV, it is preferable to use the central region of the window for extremely long-term imaging. In this work, we demonstrated that the optically cleared LF window and our stabilization strategy are suitable for spinal cord imaging in the cervical, thoracic and lumbar regions. However, it should be noted that the FOV of the intervertebral window at different parts of the spinal cord is different due to variation of the vertebral structure from cervical to lumbar region. In general, intervertebral window at the lower thoracic region provides the largest FOV in comparison with the cervical and lumbar regions^104–108^. To further extend the FOV, multiple adjacent intervertebral windows can be created on the same mouse and vertebrae can be thinned as demonstrated in a previous study^24^. Due to the limited FOV of the intervertebral LF window, this method may not be the choice for specific studies which require observing the cellular structures over a very large volume or performing severe SCI by transection or compression which could totally disrupt the LF window. Therefore, instead of replacing the previous more invasive methods allowing large FOV^12,13,20,21^, this optically cleared LF window can serve as a technique which offers unique advantages in studying scientific questions that involve in the dynamic behavior of cells right at the spinal cord dorsal surface and require long-term as well as minimal-invasive observation^18,97,100,109,110^.

## Methods

### Animal preparation

Heterozygous Cx3Cr1-GFP (B6.129P2(Cg)-Cx3cr1tm1Litt/J)^111^ transgenic mice which express EGFP in microglia were used to characterize the inflammatory activation in the spinal cord. To study axon-glia interaction, Cx3Cr1-GFP mice were crossed with Thy1-YFP (Tg(Thy1-YFP)HJrs/J)^112^ mice to generate the Thy1-YFP/Cx3Cr1-GFP transgenic line for simultaneous imaging of axon and microglia in the spinal cord. The mice of both sexes used in all experiments were 2–6 months old at the first imaging session. Mice were housed two to four animals per cage with a standard 12-h light/dark cycle in a temperature controlled environment (22–25 °C with 40–60% humidity) and had ad libitum access to food and water. Before surgery, all required tools were sterilized by autoclaving. All surfaces which would be touched during surgery were disinfected with 70% ethanol. A sterile field was created for surgery by covering the working area of benchtop with sterile drapes. Mice were anesthetized by intraperitoneal (i.p.) injection of ketamine-xylazine mixture (87.5 mg kg^−1^ and 12.5 mg kg^−1^). Hair on the dorsal surface above the thoracic spine was shaved and completely removed using depilating cream. The dorsal surface was disinfected using iodine solution. A small (~1.5 cm) midline incision of the skin was made over the T11-T13 vertebra to expose the dorsal tissue (Supplementary Fig. 4b). Muscles and tendons on both the top and sides were severed so that the spine can be held stably by clamping the vertebra with two stainless steel clamping bars on a custom-designed stabilization stage (Supplementary Fig. 4a, d, Supplementary Notes 1 and 2, Table S1). During the surgery, sterile gauze pads and sterile saline were used to control bleeding and clean the wound. A heating pad maintained at around 37° was used to keep mice warm during surgery. All animal procedures performed in this work were conducted according to the guidelines of the Laboratory Animal Facility of the Hong Kong University of Science and Technology (HKUST) and were approved by the Animal Ethics Committee of HKUST.

#### Intervertebral window

The surgical procedures for preparing conventional intervertebral windows were modified according to a previous protocol^24,25^. Briefly, the muscle tissues and tendons in the cleft between the vertebra arcs T12 and T13 were completely removed. The LF was carefully peeled using a fine-tip tweezer, while the dura was left intact. The exposed spinal cord was kept moist by irrigating with saline. To prepare the improved intervertebral window with LF, care should be taken to keep the LF unblemished when removing the muscle and tendon in the intervertebral space (Supplementary Fig. 4e). In particular, after the window with LF has been exposed, the tweezer tip should not touch the surface of the window during surgery. This is important to avoid inducing microglia activation. Moreover, when cleaning tissue with a saline flush and gauze pad, direct contact with the window surface should also be avoided. To prepare for the imaging, a coverslip was then placed on the clamping bar, and the interspace between the coverslip and the spinal cord was filled with saline or Iodixanol (Supplementary Fig. 4f, g). This interspace varied from 0.5 to 1 mm, which could be slightly adjusted via spine clamping, allowing convenient administration of various treatments, such as vital dyes or drugs^113^. But the penetration efficiency of applied molecules through the LF and meninges should be carefully characterized. After imaging, the medium below the coverslip was removed and the surgical area was carefully cleaned using saline and gauze pads. The top area of the surgical window was then covered by liquid Kwik-Sil (World Precision Instruments) to protect it (Supplementary Fig. 4j). After the Kwik-Sil got cured (~3 min), the skin on the surgical site was sutured and covered with burn cream (Betadine) to protect from infection. Buprenorphine (0.1 mg/kg) was administered subcutaneously for post-operative analgesia. Mice were placed on a heating pad until they recovered fully from anesthesia. For reimaging through the same intervertebral window with LF, the sutured skin was reopened and the covering Kwik-Sil gel was removed. Tissues adhering to the side of the T11-T13 vertebra were detached to enable stable clamping of the spine. If reimaging was performed within four days of the initial surgery, granulation tissue had not formed at the surgical site. Therefore, we only need to clean the window surface by flushing saline and remove loose tissue debris from the surface. With the growth of granulation tissue accompanied by angiogenesis and fibroplasia, the tissues on the surface of the surgical site should be peeled off to expose the LF, which can be easily distinguished from the newly generated tissues by its tough collagenous structures. In addition, the laminae and processes of two vertebrae around the window should always be scraped clean without tissue adhesions. The procedures for imaging and post-imaging preparations are the same as previously described. Surgical preparation of the intervertebral LF window in the cervical (C3-C7) and lumbar (L1-L2) regions is similar to the T12-T13 lower thoracic region, except that the spine clamping bars were redesigned for cervical spinal cord stabilization (Supplementary Fig. 9a).

#### Dorsal column crush

The spinal cord dorsal column crush was conducted following previous protocols^77,114^ with slight modifications. Briefly, T12 laminectomy was performed to expose the spinal cord using Dumont #2 Laminectomy forceps. Two small holes were made in the dura with a 30-gauge needle symmetrically around 0.5 mm lateral to the midline. A dorsal hemi-crush injury was made by inserting the modified Dumont #5 forcep through the two small holes approximately 0.6 mm into the dorsal spinal cord and squeezing with pressure for 5 s, and repeating three times.

### Multimodal NLO microscopy

The setup of our multimodal NLO microscope is shown in Supplementary Fig. 1. An integrated optical parametric oscillator (OPO, picoEmerald S, APE) was used as the light source for SRS imaging. It consists of a Stokes beam (1031 nm) and pump beam (tunable from 780 nm to 960 nm) with 2 ps pulse duration and 80 MHz repetition rate. The intensity of the Stokes beam was modulated at 20 MHz by a built-in electro optical modulator. The pump beam was combined with the Stokes beam using a dichroic mirror (D1) inside the picoEmerald S. A femtosecond Ti:sapphire laser (Chameleon Ultra II, Coherent) tuned to 920 nm was used as the laser source for exciting two-photon fluorescence and generating second harmonic generation signals. The fs beam was rotated from horizontal to vertical polarization by a half-wave plate(SAHWP05M-1700, Thorlabs) and then combined with the ps beam by a polarizing beam splitter (CCM1-PBS252/M, Thorlabs). The ps beam and fs beam were collimated and magnified by a pair of achromatic doublets to match the 3 mm Galvo XY-scan mirror (6215H, Cambridge Technology). The Galvo mirror and the rear pupil of the objective lens (XLPLN25XSVMP2, 25×/1.05 NA, 2-mm working distance, Olympus) were conjugated by a telecentric scan lens L5 (SL50-CLS2, Thorlabs) and an infinity-corrected tube lens L6 (TTL200-S8, Thorlabs). The laser beam was expanded by the scan and tube lens to fill the back aperture of the objective.

For SRS imaging, the backscattered pump beam collected by the objective was reflected by a polarizing beam splitter (CCM1-PBS252/M, Thorlabs) and directed to a large area (10 mm$\times$10 mm) Si photodiode (APE). A dichroic short-pass filter D3 (69–206, short-pass at 700 nm, Edmund) was used to separate the SRS detection path from the fluorescence detection path. A filter set (Fs1) including a short-pass filter (86–108, short-pass at 975 nm OD4, Edmund) and a band-pass filter (FF01-850/310, Semrock) were placed before the photodiode to completely block the Stokes beam. The output of the photodiode was then fed into a lock-in amplifier (LIA) for signal demodulation and amplification to obtain highly sensitive detection of stimulated Raman loss (SRL).

For two-photon imaging, the polarizing beam splitter above the objective was replaced by a dichroic beam splitter D2 (FF665-Di02, Semrock) to reflect the TPEF and SHG signal to the photodetection unit. An interchangeable dichroic beam splitter D4 (FF488-Di01-25×36 or FF518-Di01-25 × 36, Semrock) was placed after D3 to separate the fluorescence into two current photomultiplier (PMT) modules (H11461-03 and H11461-01, Hamamatsu). Two filter sets Fs2 (FF01-715/SP-25, Semrock; FF01-525/50, Semrock or HQ620/60X, Chroma) and Fs3 (FF01-720/SP-25, Semrock; FF01-525/50, Semrock or HQ440/80 M, Chroma) were placed before the PMTs to reject the excitation beam and transmit fluorescence. The output currents of the two PMTs were then converted to voltage by two current amplifiers (SR570, Stanford research). The outputs of the two current amplifiers and LIA were then fed into a multifunction acquisition card (PCIe-6363, National Instrument) to reconstruct the image. For spectral characterization of emitted TPEF, the dichroic mirror D4 was switched to 665dcxr (Chroma) to reflect fluorescence onto a fiber-based spectroscopic detection module. The reflected fluorescence was filtered by a short pass filter (SP01-633RU-25, Semrock) and coupled into a fiber bundle before being directed to a multispectral detection system consisting of a spectrograph (455 ~ 650 nm) equipped with a 16-channel PMT module (PML-16-C-0, Becker & Hickl). This detection system allows spectral measurements for each pixel of the TPEF image with a 13-nm spectral resolution. All the hardware was controlled by a custom-written C# program to acquire two-photon and SRS images.

The hyperspectral SRS sweeping mode was used to acquire the SRS spectra of solutions and tissues in the fingerprint and C-H stretch region. First, temporal overlapping calibration of the pump and Stokes beams at the fingerprint and C-H vibration regions was performed by adjusting a built-in delay stage based on the SRS signal of 6 μm polystyrene beads (Polysciences, Inc., Warrington, PA), Olive oil and heavy water (99% pure, D2O) at their specific Raman peaks. Since solution samples are homogenous with little scattering, to achieve SRS imaging of solutions in an epi-detection configuration, a piece of folded tissue paper was stuck to the bottom of the slide to backscatter the SRS signals. The wavelength of the pump beam was sequentially tuned with 0.3-nm steps by the program through a serial communication port. For Iodixanol SRS imaging, the Lyot filter inside the laser was adjusted to fast tune the pump wavelength from 891.1 nm (1523 cm^−1^, on-resonant) to 893 nm (1500 cm^−1^, off-resonant). By synchronizing the Lyot filter to the frame trigger, a pair of “on-resonant” and “off-resonant” images could be acquired with less than 3 s switching time. The final Iodixanol image was obtained by subtracting the off-resonant signals from the on-resonant signals.

### In vivo imaging

Before each imaging session, the mouse received a retro-orbital intravenous injection of 100 ${{{{{\rm{\mu}}}}}}$l Texas Red dextran (70 kDa, 1 mg/100 ul in saline, Invitrogen) to label blood vessels when necessary. The stabilization stage securing the mouse was placed on a five-axis stage beneath our customized microscope. The five-axis stage allows three-axis translation and $\pm {5}^{^\circ }$pitch and roll flexure motion. To reduce the motion artifacts caused by breathing, the mouse head was secured by two head bars and the mouse body was slightly suspended by lowering the holding plate to allow space for chest movement during breathing (Supplementary Fig. 4a, d). The holding plate should not be lowered too far away from the mouse body in order to keep good contact between the heating plate and the mouse. The mouse’s spinal cord was aligned perpendicular to the objective axis by adjusting the roll and pitch angles of the stage guided by the bright-field image (4$\times$ objective, 0.16 NA, Olympus). Since the spinal cord has a natural curvature, in order to align the sample surface precisely during NLO imaging over a large FOV, the angle needs to be finely adjusted for each small sub-region guided by the TPEF signal of each FOV. The femtosecond laser was tuned to 920 nm for TPEF excitation of GFP, YFP or Texas Red. First, a 10$\times$ objective lens (NA = 0.45, Nikon) was used to obtain an image of the entire intervertebral window as a roadmap for navigating between imaging sessions. Then a 25$\times$ water immersion objective (NA = 1.05, Olympus) was used for high-resolution two-photon and SRS imaging of the target area. For two-photon imaging with 10$\times$ and 25$\times$ objectives, the post-objective power ranged from 40 to 65 mW and 10 to 50 mW, respectively, depending on the clarity of the intervertebral window. For SRS imaging with 25$\times$ objective, the post-objective power of pump and Stokes beam ranged from 50 to 70 mW and 60 to 80 mW, respectively. During imaging, the holding plate was heated to 37 °C to keep the mouse warm. Ketamine-xylazine (43.75 mg kg^−1^; 6.25 mg kg^−1^)) were supplemented when necessary.

### Optical clearing by Iodixanol

Iodixanol/OptiPrep (D1556, Sigma-Aldrich) was purchased as a 60% w/v solution of iodixanol in sterile water and prepared with various concentrations by diluting the 60% w/v stock solution in sterile phosphate buffered saline (PBS). Stock solutions were directly used for optical clearing with 60% iodxianol (w/v). To achieve optical clearing of the IWLF, Iodixanol was applied and supplemented hourly. Imaging was usually started 10 min after every Iodixanol administration when the optical clearing effect reached a plateau.

### Histology

Mice were deeply anesthetized and then perfused transcardially with 20 ml PBS to wash out the blood and 20 ml 4% (w/v) PFA (Sigma-Aldrich) for fixation. Spinal cord segments (~1 cm) at the surgical and control region were dissected out and immersed in 15% (w/v) sucrose PBS solution for 12 h before further dehydration in 30% sucrose PBS solution. After sedimentation, samples were frozen at −80 °C and then cut to 50 um-thick sagittal sections on a CryoStar NX70 Cryostat (Thermo Scientific). The GFP-labeled microglia cells located less than 50 $\mu$m below the dorsal surface were imaged for morphological analysis.

### Laser axotomy

Laser axotomy is achieved by a highly localized nonlinear process based on multi-photon ionization and plasma-mediated ablation^115^. To perform imaging-guided laser axotomy, a femtosecond laser tuned to 920 nm was focused on the targeted axon for 1–4 s with an average power of 250 mW. The lesion caused by laser ablation can be visualized and quantified by the newly generated fluorescence^115,116^ or SRS signal of the spinal cord.

### Image processing and analysis

Since in vivo spinal cord imaging would be affected severely by motion artifacts caused by breathing and heartbeats, it is necessary to perform image registration to acquire stable images. For multimodal NLO imaging, three-dimensional (3D) optical sectioning was performed to obtain images at different depths. To reduce intra-frame distortion, 10 frames (512$\times$512 pixels) were acquired per slice with a 2-Hz frame rate. Single-channel 3D image registration was carried out as follows. First, image registration was performed on the sequential frames for each slice using the ‘StackReg’ plugin^117^ in Fiji software^118^. Then the registered frames were averaged to form a target image used for the next step of registration. Using the ‘bUnwarpJ’ plugin^119^ in Fiji, each raw image frame was then registered individually to the target image. The registered frames were then averaged to obtain the final slice for each depth with minimal motion artifacts and improved signal-to-background ratio. Registration between slices was then performed using the ‘StackReg’ plugin to construct the 3D image stack. For two-channel 3D image registration, since each pair of the two-channel images was acquired simultaneously with the same deformation, they should be registered using the same transformation parameters. During ‘bUnwarpJ’ registration, the transformation information for each frame of the single-channel images was recorded and then applied to the corresponding frames of another channel. To co-localize SRS image and fs laser-excited TPEF image, we captured the ps laser-excited TPEF images simultaneously with the SRS image, which were then used as reference images for SRS and fs TPEF image registration. The ‘MultiStackReg’ plugin in Fiji was used for multichannel stack registration using the same transformation parameters. All these registration procedures were implemented in the Fiji macro programming language. To acquire the large-FOV images of the intervertebral window as shown in Figs. 1c and 4a, Supplementary Fig. 4a, Supplementary Fig. 20a, Supplementary Fig. 21 and Supplementary Fig. 22, the sub-images were stitched using the ‘Pairwise stitching’ plugin^120^ in Fiji. All the two-photon fluorescence images were mean filtered with 1-pixel radius and normalized to saturate 1% (for GFP images of microglia) or 0.3% (for other fluorescence images except microglia) pixels after background subtraction. Image enhancement was achieved by normalizing the whole image to a smaller value corresponding to the enhancement ratio.

To calculate the density of GFP labeled macrophages in the epidural space (Supplementary Fig. 6d) and meninges (Supplementary Fig. 15c), GFP images were stacked as a maximum z projection and image contrast was adjusted to saturate 0.3% pixels. The GFP maximal projection images were then converted to a bit-map using FIJI ‘Threshold’ plugin with ‘Default’ auto thresholding method. The macrophage density was then calculated as the ratio of macrophage area to the FOV (300 $\times$ 300 $\mu m$) area.

To characterize the image contrast, fluorescence images were stacked as a maximum z projection. In each FOV, microglia with visible cell bodies were randomly selected for contrast characterization. An intensity profile was plotted through the center of the microglia cell body. The peak value of the profile is represented as${I}_{0}$. The background value B is defined as the averaged intensity value over a 5$\,\mu$m region along the intensity profile at 10–20 $\mu$m away from the intensity peak^121^. The image contrast can be calculated as^20^:

1 $$\frac{{I}_{0}-B}{{I}_{0}+B}$$

A contrast value of 0 represents no contrast while 1 represents noiseless contrast.

For quantification of microglia ramification index and process endpoints, microglia with intact and clear morphology (contrast $\ge 0.97$) (Supplementary Fig. 17) in each FOV were randomly selected for morphological quantification without bias. The analysis was performed using Fiji and MATLAB (The MathWorks) scripts based on the published methods^40,41^ with small modifications. Briefly, binary images of individual microglia were first acquired and saved as independent files as previously described^40^. Microglial binary images were skeletonized using the ‘bwskel’ MATLAB function and the endpoints of each microglial skeleton were counted using the ‘bwmorph’ MATLAB function. To quantify the ramification index, the MATLAB functions ‘bwarea’ and ‘bwperim’ (8-connected neighborhood) were used to acquire the area and perimeter value of each cell. The ramification index is then calculated as^40^:

2 $$(perimeter/area)/[2\cdot{(\pi/area)}^{1/2}]$$

For analysis of microglia aggregation and axonal degeneration, a dot rectangular region of interest was outlined along the axonal degeneration path to quantify the spatial distribution of the microglial aggregation after laser axotomy (Fig. 4a). The maximal z projection images of axon and microglia in the rectangle area were mean filtered with 1-pixel radius and converted to a bit-map with FIJI ‘Threshold’ plugin by setting a lower threshold level to be 8% of the maximal value for microglia image and 11% for axon image. The aggregation size of the microglia was calculated as the sum of the GFP fluorescence signals in the bit-mapped area normalized by the fluorescence intensity of surrounding uninjured axons. The axonal diameter was measured based on the YFP fluorescence signals along the degeneration path.

For microglia-nodes of Ranvier contact analysis, the positions of the nodes of Ranvier on the axon were first confirmed by merging the myelin SRS image and axon TPEF image. Axon images were then merged with microglia images to visualize microglia contacts with the nodes of Ranvier. To reduce noise, images were smoothed after background subtraction. Contacts were defined as the 3D colocalization of the fluorescence of microglia and the node of Ranvier. Wrapping contact was identified when the nodes of Ranvier were totally enveloped by microglia processes.

3D reconstruction of the in vivo multimodal image stacks was performed with Imaris (Bitplane AG, Zurich, Switzerland).

### Statistical analysis

Statistical analysis and data visualization were performed using GraphPad Prism 7 software. All the data are presented as mean $\pm$ s.e.m. and $\alpha =$0.05 for all analyses. P values for ordinary one-way ANOVA with Dunnett’s multiple comparison test (for normally distributed data) or Kruskal-Wallis test (for non-normally distributed data) are given on the figures. Data normality was checked using the Shapiro-Wilk normality test. No statistical methods were used to predetermine sample sizes, but our sample sizes were similar to reported studies of chronic spinal cord imaging^20,21^.

For the calculation of the success rate of window preparation, since the LF window for each imaging session is prepared following the same surgical protocol, each session can be approximately regarded as an independent test for the success rate of LF window preparation which has two potential outcomes (“success” and “failure”). Statistically, the outcomes of all the LF window preparations performed in each mouse form a sequence of independent Bernoulli trials following a negative binomial distribution^59,60^, and each sequence ends when the outcome is “failure” or activation of microglia. The probability of activating microglia, *p*, is calculated based on the observations of the sequences from multiple mice used in the study^59,60^:

3 $$p=\frac{N}{N+{\sum }_{i=1}^{N}{k}_{i}}$$

where *N* denotes the number of mice used in the study, and ${k}_{i}$ denotes the number of successful surgeries observed for each mouse. The rate of failure is *p* and of success is (*1 − p*).

### Reporting Summary

Further information on research design is available in the Nature Research Reporting Summary linked to this article.

## Supplementary information

Supplementary Information

Supplementary note 2

Supplementary Movies

Reporting Summary

## Data Availability

All the data supporting the findings of this study are available within the paper and its supplementary information files. Source data are provided with this paper. Other extended data figures are available from the corresponding author upon request. Owing to the size of the datasets, they are not available on a public data repository. Source data are provided with this paper.

## Source data

Source Data

## Code availability

The MATLAB scripts for calculation of microglial ramification index and process endpoints are available online at “https://github.com/JaneWuhkust/Microglial-morphological-analysis”.
