# Publisher Correction: Long-term in vivo imaging of mouse spinal cord through an optically cleared intervertebral window

**DOI:** 10.1038/s41467-022-31648-y

**Published:** 2022-07-07

**Authors:** Wanjie Wu, Sicong He, Junqiang Wu, Congping Chen, Xuesong Li, Kai Liu, Jianan Y. Qu

**Affiliations:** 1 Department of Electronic and Computer Engineering, The Hong Kong University of Science and Technology, Clear Water Bay, Kowloon, Hong Kong, P. R. China; 2 Department of Biology, School of Life Sciences, Southern University of Science and Technology, Shenzhen, China; 3 Division of Life Science, The Hong Kong University of Science and Technology, Clear Water Bay, Kowloon, Hong Kong, P. R. China; 4 State Key Laboratory of Molecular Neuroscience, The Hong Kong University of Science and Technology, Clear Water Bay, Kowloon, Hong Kong, P. R. China; 5 Center of Systems Biology and Human Health, The Hong Kong University of Science and Technology, Clear Water Bay, Kowloon, Hong Kong, P. R. China; 6 Molecular Neuroscience Center, The Hong Kong University of Science and Technology, Clear Water Bay, Kowloon, Hong Kong, P. R. China

**Keywords:** Multiphoton microscopy, Cellular neuroscience, Optical imaging

Correction to: *Nature Communications* 10.1038/s41467-022-29496-x, published online 12 April 2022.

The original HTML version of this Article was updated shortly after publication because the previous HTML incorrectly linked to a Supplementary Figures file.

