# Supplementary material for: Long-term in vivo imaging of mouse spinal cord through an optically cleared intervertebral window
Source: Nat Commun. 2022 Apr 12;13:1959. doi: 10.1038/s41467-022-29496-x (PMC9005710; doi:10.1038/s41467-022-29496-x)
Supplement: Supplementary file 1 — Supplementary Information [file 41467_2022_29496_MOESM1_ESM.pdf]

Supplementary Information

Long-term *in vivo* imaging of mouse spinal cord through an optically cleared intervertebral window

Wu et al.

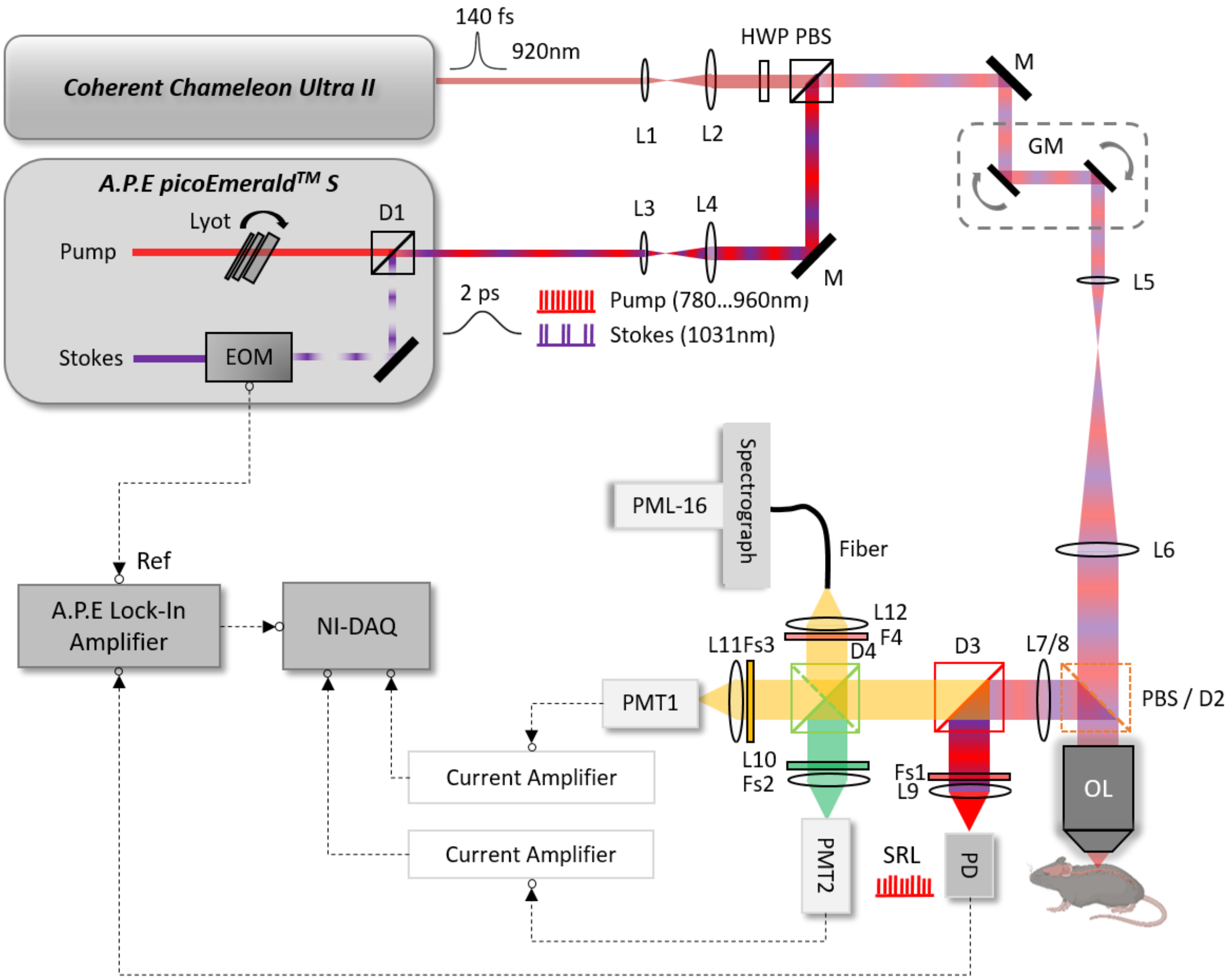

Supplementary Figure 1. Schematic diagram of the multimodal NLO microscope system. EOM: electro-optical modulator; HWP: half-wave plate; PBS: polarizing beam splitter; L1- L12: lenses; OL: objective lens; D1-D4: dichroic mirrors; Fs1-Fs3: filter sets; F4: filter; M: mirror; GM: galvo scanning mirrors; PD: photodiode; SRL: stimulated Raman loss; PMT1-2: photomultiplier tubes; PML-16: 16-channel photomultiplier tube; NI-DAQ: National Instruments data acquisition device.

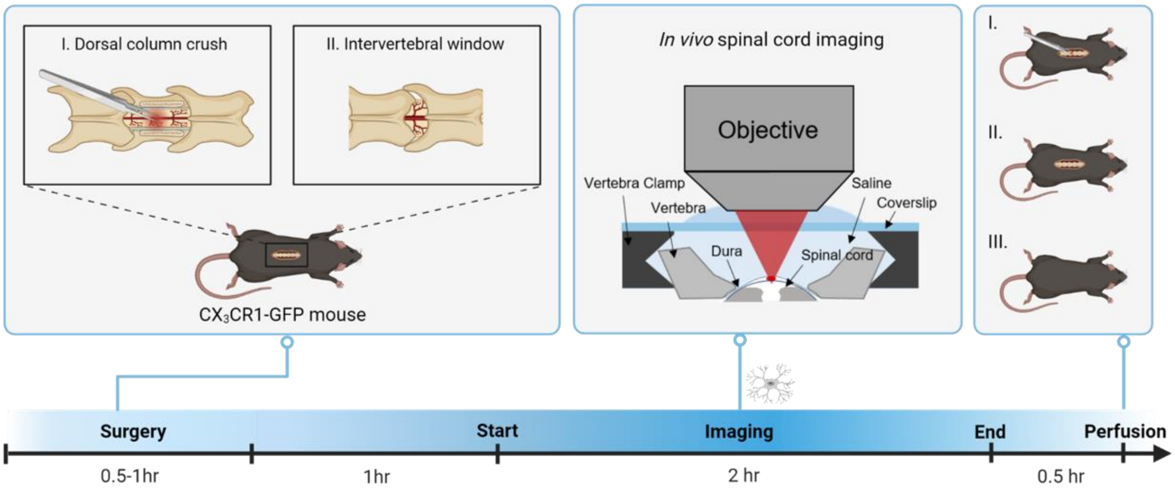

Supplementary Figure 2. **Experimental design for characterization of inflammation induced by the surgical preparations.** Group I, mice that underwent laminectomy and dorsal column crush (DCC); Group II, mice with spinal cord exposed in the intervertebral gap and only dura left above; Group III, mice that didn't undergo surgery used as a negative control group (N.C.); 3 mice per group. Figure created using BioRender (https://biorender.com/).

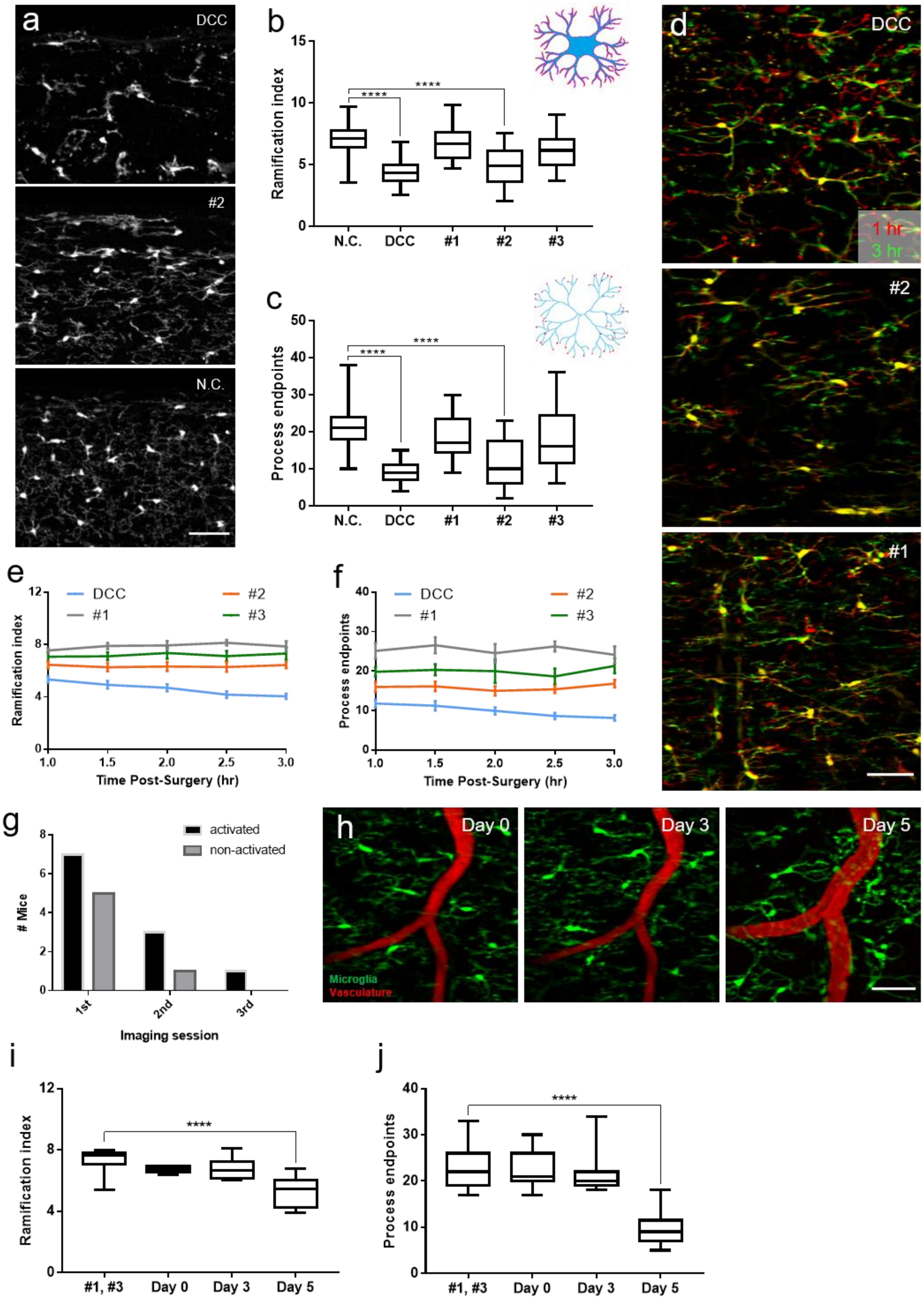
a
DCC
#2
N.C.
b
Ramification index
N.C.
DCC
#1
#2
#3
****
c
Process endpoints
d
1 hr
3 hr
e
Ramification index
Time Post-Surgery (hr)
f
Process endpoints
Time Post-Surgery (hr)
g
activated
non-activated
Mice
1st
2nd
3rd
Imaging session
h
Day 0
Day 3
Day 5
Microglia
Vasculature
i
j
#1, #3

Supplementary Figure 3. **Microglia showed high activation probability under the conventional intervertebral window preparation** (a) Representative two-photon fluorescence images of 50-um-thick longitudinal spinal cord slices from the mice expressing EGFP in microglia. DCC: image of slice from dorsal column crush group; #2: image from Mouse#2 in the intervertebral window group; N.C.: image from negative control group. Scale bar, 50 μm. (b, c) Evaluation of microglia ramification index (b) and number of process endpoints (c) of spinal cord fixed slices from the three groups. N.C.: negative control group; DCC: dorsal column crush group; #1-3: three mice in the intervertebral window group. n $\geq$ 20 microglial cells were analyzed from 6-8 slices per mouse, three mice per group. (d) Representative *in vivo* superimposed images of microglia at 1 hour and 3 hours post-surgery with three independent repetitions of three mice. Scale bar, 50 μm. (e, f) Changes of the microglia ramification index (e) and process endpoints (f) during two-hour *in vivo* imaging of intervertebral window and DCC group. Images of the same region were taken at an interval of 30 minutes. 6-8 microglial cells were analyzed per time point per mouse. (g) Binary plot of microglial activation statistics during longitudinal imaging through a conventional intervertebral window. Microglial activation was observed in 11 out of 17 imaging sessions of 12 mice, determined by comparing the ramification index and number of process endpoints measured in each session with the same parameters from the mice (#1 and #3 in (e-f)) without activation of microglia. The longitudinal study was terminated when microglial activation was observed. (h) Longitudinal imaging of microglia (green) and vasculature (red) through a conventional intervertebral window at indicated times. Blood vessels were labeled with Texas Red dextran. Scale bar, 50 μm. (i, j) Ramification index (i) and process endpoints (j) of microglial cells shown in (h) and comparison with the the same parameters from the mice #1 and #3 in (e-f) without activation of microglia. Kruskal-Wallis test: ****P $\leq$ 0.0001; The boxplots are shown with median, upper and lower quartiles and maximum and minimum values. Error bars, s.e.m. Source data are provided as a Source Data file.

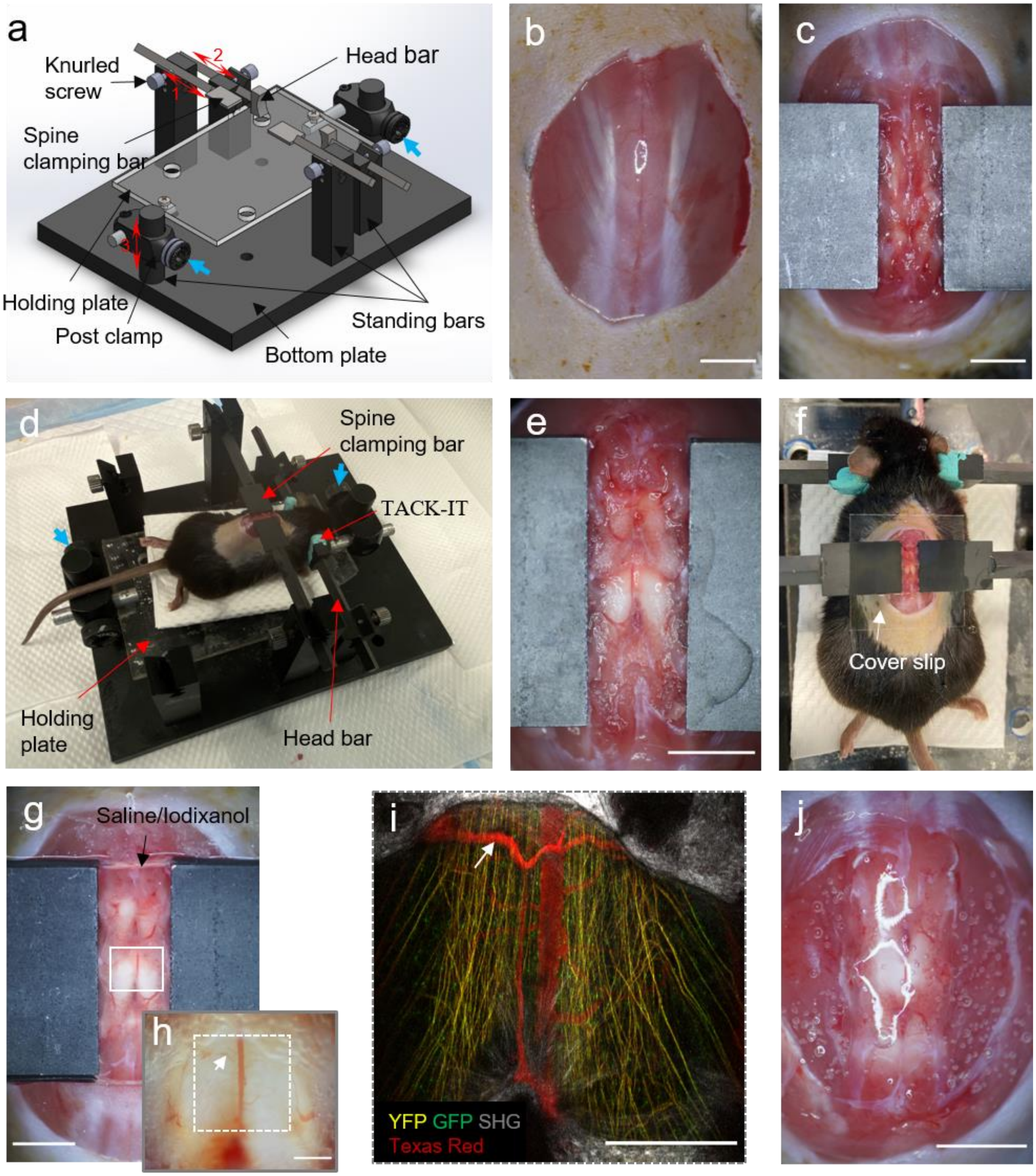

Supplementary Figure 4. Surgical procedures of intervertebral LF window. (a) A custom-designed stage for spinal cord stabilization during surgery and imaging. The stage consists of two spinal cord clamping bars to secure the spinal column. The two head bars are used to stabilize the mouse's head by holding its skull, which reduces motion artifacts. Both the head bars and spine clamping bars can move

along the direction indicated by the red arrow 1 and 2. Their positions can then be fixed with knurled screws in the standing bars. A holding plate with adjustable height (along vertical direction indicated by the red arrow 3) was used to slightly suspend the mouse body, allowing space for chest movement during breathing. The screws indicated by blue arrows are used to fix the position of the holding plate. (b-j) The procedure of the first surgical preparation of the LF window. (b) A small (~1.5cm) midline incision of the skin was made over the T11-T13 vertebrae and the skin was partly cut to expose the dorsal tissue. Scale bar, 3 mm. (c) Muscles as well as tendons both on both the top and sides were severed to clean the bone. Scale bar, 3 mm. (d) The mounting scheme of mouse on the stabilization stage. The green TACK-IT (FABER-CASTELL) were attached to the tip of head bar for better fitting to the shape of skull and release of mechanical stress to head. Blue arrows indicate the same screws shown in (a). A heating plate (not shown) was inserted between the tissue paper and the holding plate to keep mouse warm during surgery and imaging. (e) Tissues attached to the vertebra were further resected. Muscle tissues and tendons in the cleft between the vertebra arcs T12 and T13 were removed to expose the intervertebral window. Scale bar, 3 mm. (f-g) A coverslip (22 mm $\times$ 22 mm) was placed on the clamping bar and the interspace between the coverslip and spinal cord was filled with saline or Iodixanol solution. Scale bar, 3 mm. (h) Magnified bright-field image in the box region of (g). Blood vessels (white arrow) in the epidural space can be visualized in the bright-field image. Scale bar, 500μm. (i) Maximal projection of the TPEF and SHG image stack of the dashed box region in (h). The same blood vessel in the epidural space is also indicated by the white arrow. Blood vessels were labeled with Texas Red dextran by retro-orbital intravenous injection. Scale bar, 500 μm. (j) After imaging, liquid Kwik-Sil (World Precision Instruments) was applied on top of the surgical area covering the window for protection. Scale bar, 3 mm.

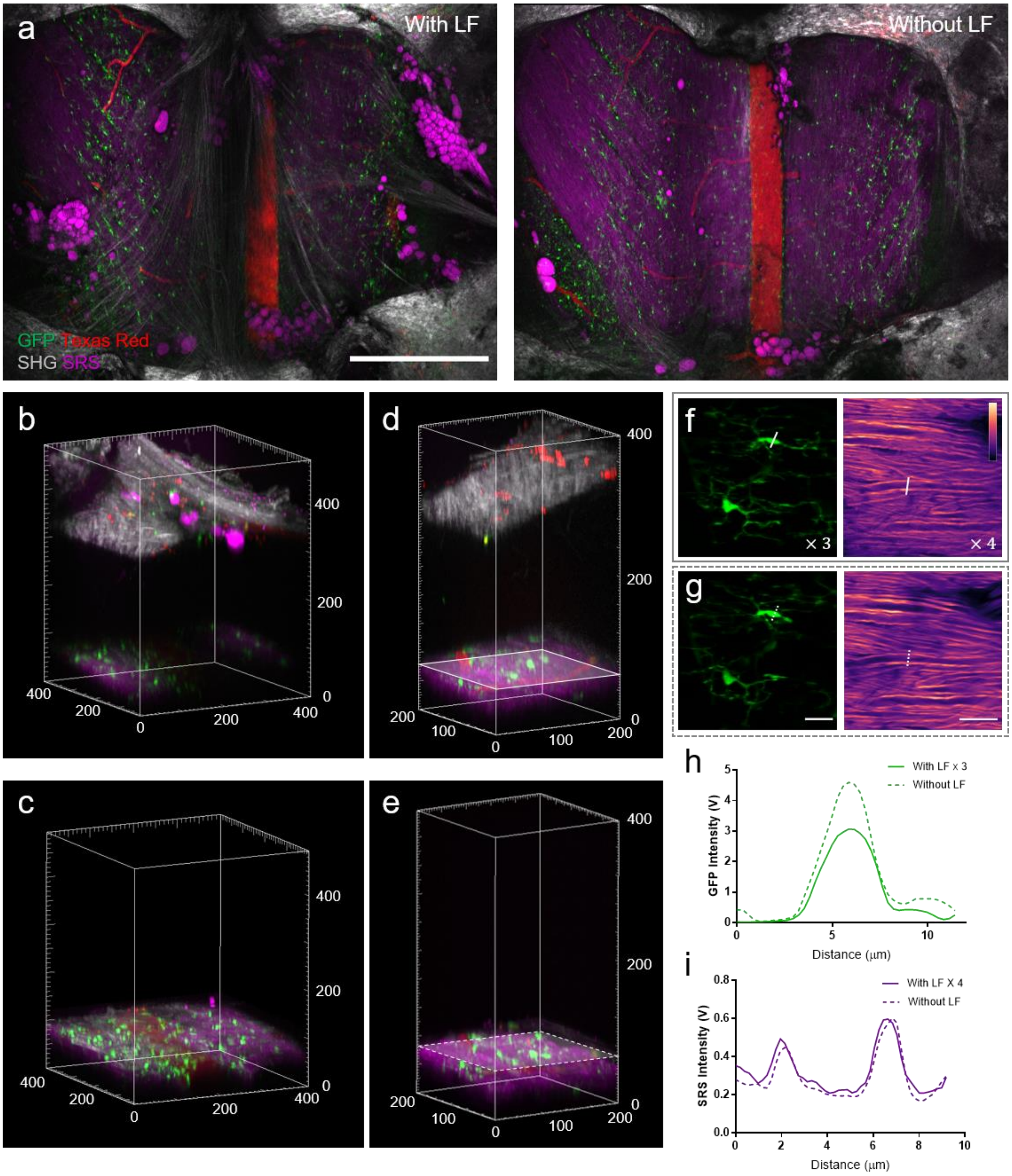

Supplementary Figure 5. **Optical transparency and homogeneity of the intervertebral window affected by LF layer.** (a) Projection of an *in vivo* multimodal image stack of the intervertebral window before and after removal of ligamentum flavum. Scale bar, 500 μm. (b-e) 3D reconstruction of the

multimodal imaging stacks at the location where spinal cord imaging is most affected by the existence of ligamentum flavum (b,c) and where imaging is less affected (d,e). Spinal cord *in vivo* imaging is compared at the same location before (b,d) and after (c,e) removal of ligamentum flavum. In (a-e): Green: GFP labeled microglia; Red, blood vessels and immune cells labeled with Texas Red dextran; Gray, second harmonic generation (SHG) signals of collagen and other connective tissues; Magenta, stimulated Raman scattering (SRS) signals of adipose tissue and myelin. (f, g) Maximal projection of TPEF microglia images (left) and SRS myelin images (right) at the same location of (d-e) before (f) and after (g) ligamentum flavum removal. Scale bar, 20 μm. Images in (f) and (g) are normalized to the same value and images in (f) were enhanced 3 and 4 fold digitally as indicated for better visualization. (h, i) Signal profile along the solid and dashed lines in (f) and (g) for a comparison of the fluorescence and SRS intensity before and after ligamentum flavum removal. SRS images are taken at Raman shift of 2863.5 cm^{-1}. Source data are provided as a Source Data file.

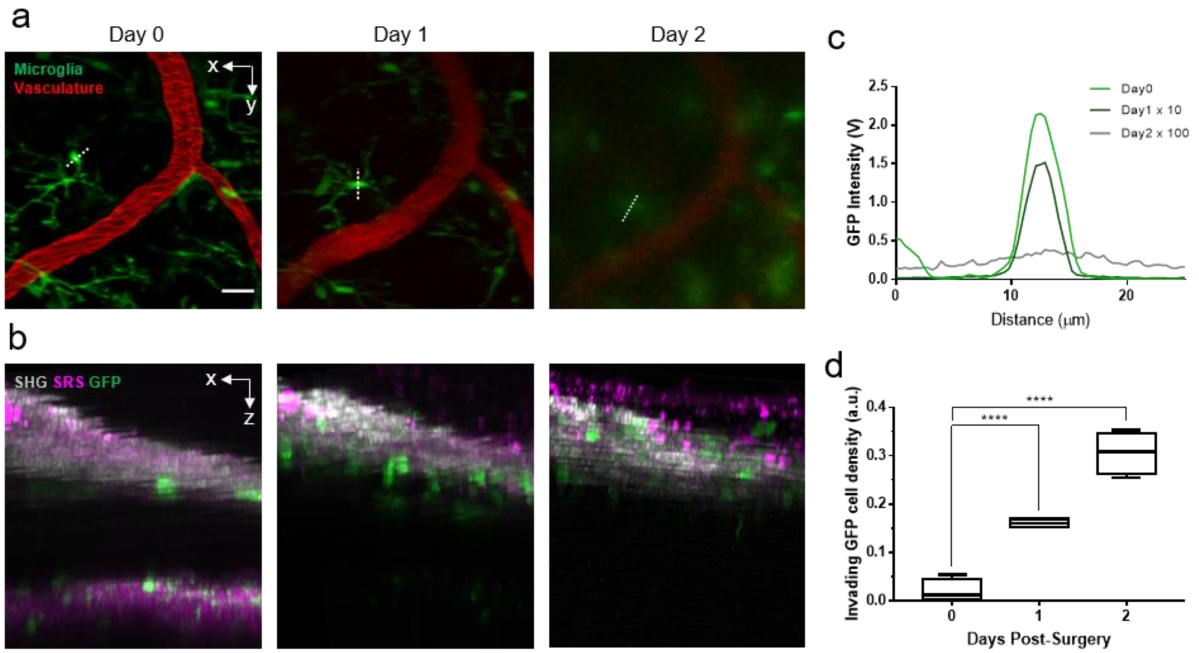

Supplementary Figure 6. **Signal intensity of *in vivo* spinal cord imaging decreases over days through LF window.** (a, b) Maximal x-y projection of microglia (green) and vasculature (red) images (a) under LF window and the corresponding x-z view (b) of the intervertebral window at indicated times. TPEF and SRS intensity in (a) and (b) are individually normalized for better visualization. Red, blood vessels labeled with Texas Red dextran; Gray, SHG signals of ligamentum flavum and meninges; Magenta, SRS signals of myelin and other tissues acquired at Raman shift of 2863.5 cm^{-1}. Scale bar for x,y,z dimensions, 20 μm. (c) Signal profile along the dashed lines in (a) for a comparison of the fluorescence intensity at indicated times. (d) Density of the invading GFP cells in the same imaging volume of 300 μm $\times$ 300 μm $\times$ 100 μm increases with time. One-way ANOVA: **P = 0.005, ****P≤0.0001. 1-2 ROIs measured for each mouse, three mice for each time point. The box plot is shown with median, upper and lower quartiles and max and min values. A.u., arbitrary units. Source data are provided as a Source Data file.

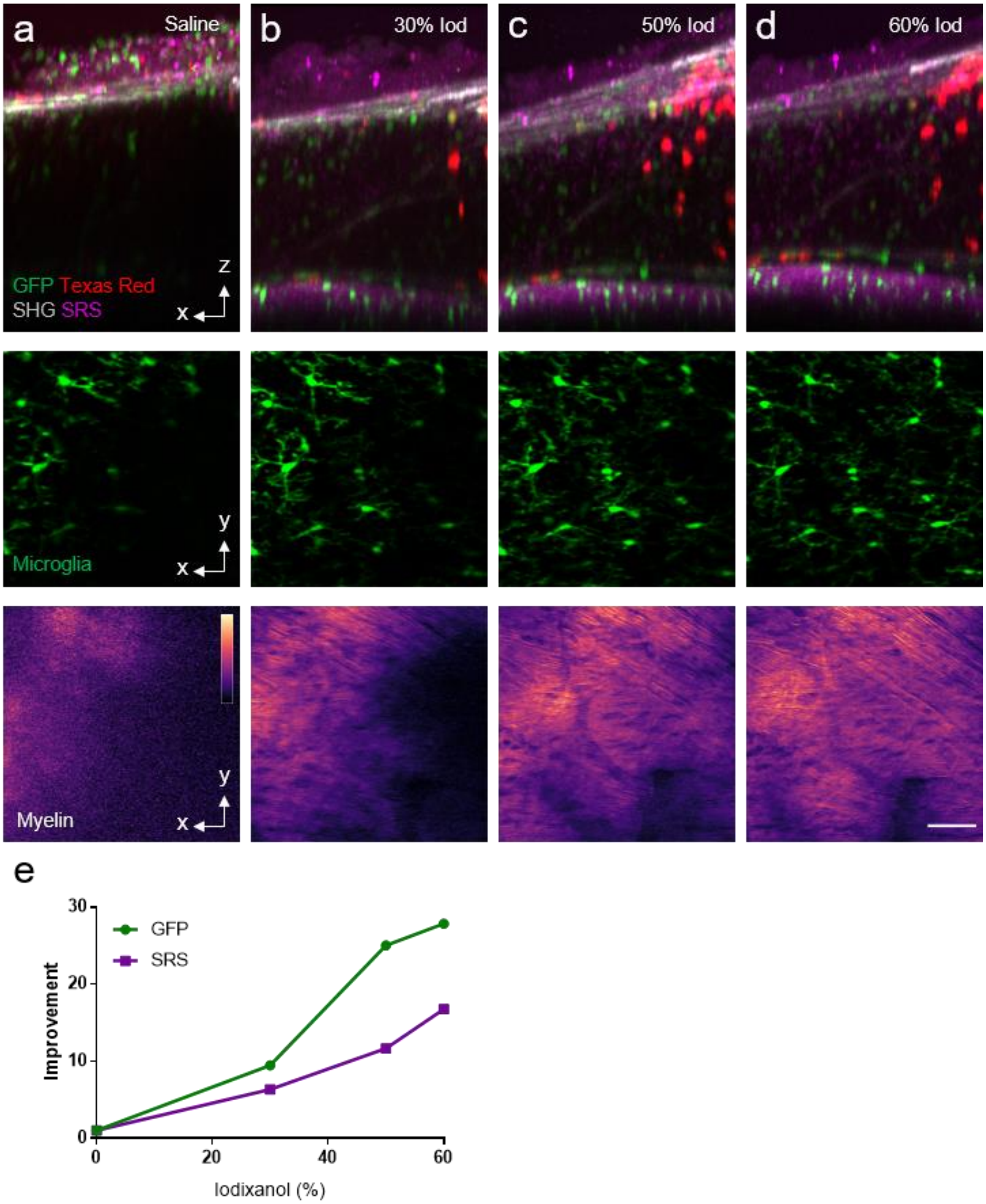

Supplementary Figure 7. Effect of optical clearance as a function of Iodixanol concentration. (a-d) Maximal x-z projection of a multimodal image of the intervertebral window and the maximal x-y projection image of microglia and myelin under the application of saline (a), 30% (b), 50% (c) and 60% (d) Iodixanol.

Green: GFP labeled microglia and other immune cells; Red, blood vessels and immune cells labeled with Texas Red dextran; Gray, SHG signals of ligamentum flavum and meninges; Magenta, SRS signals of myelin and other tissues acquired at Raman shift of 2863.5 cm^{-1}. Scale bar for x,y,z dimensions, 50 μm. (e) Quantification of the improvement in the microglia GFP and myelin SRS signal obtained by using Iodixanol at different concentrations. Source data are provided as a Source Data file.

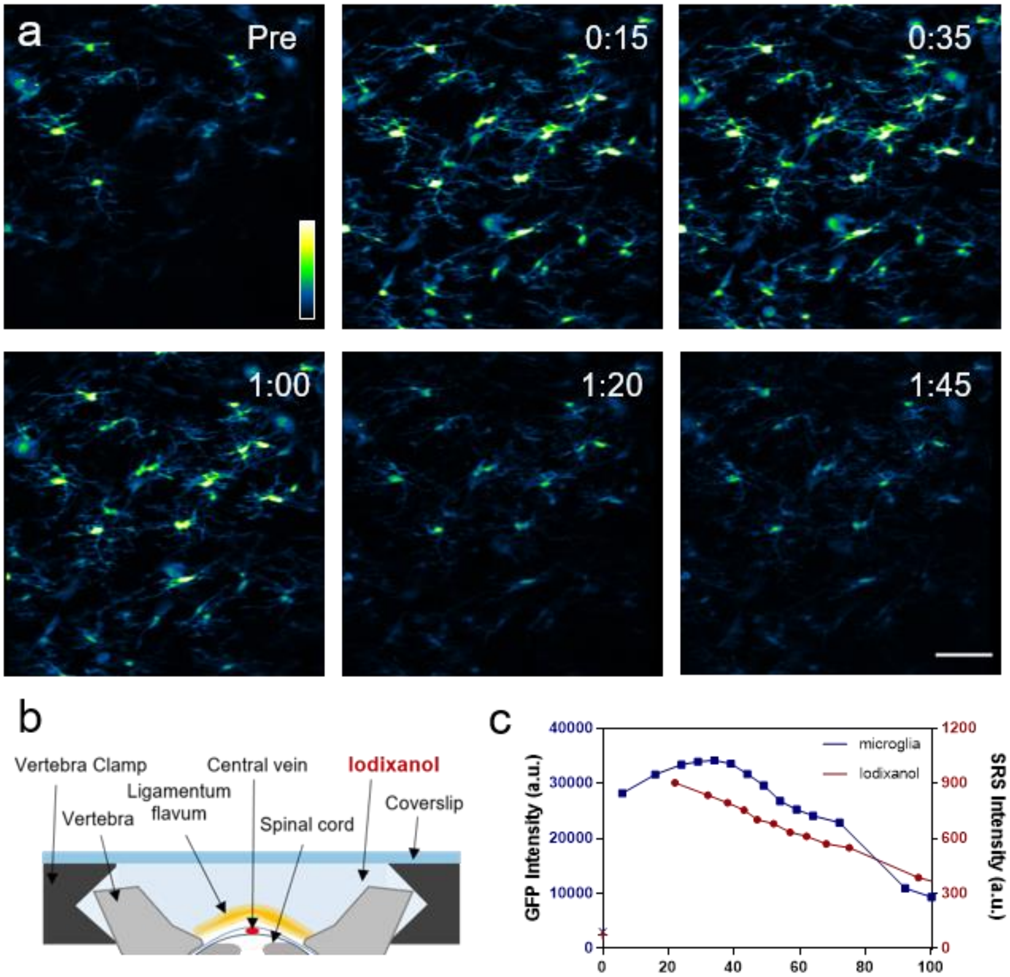

Supplementary Figure 8. Iodixanol washout in LF window as a function of time. (a) Maximal projection of microglia TPEF image stack at the indicated time before and after Iodixanol application. Images are normalized to the same value. Time is presented as hr:min. Scale bar, 50 μm. (b) Schematic diagram of the intervertebral window setup for optical imaging. Iodixanol fills the space between the spinal cord and coverslip. (c) GFP intensity of microglia and SRS intensity of Iodixanol as functions of time after the application of Iodixanol. Signals before Iodixanol application are denoted as cross symbols at time 0. GFP intensity is the average intensity of the brightest 0.1% pixels in the microglia maximal projection images. Iodixanol SRS images are taken 10 μm above the ligamentum flavum at Raman shift of 2943 cm^{-1}, corresponding to its Raman peak in the C-H region. SRS intensity is calculated as the average intensity of the image. A.u., arbitrary units. Source data are provided as a Source Data file.

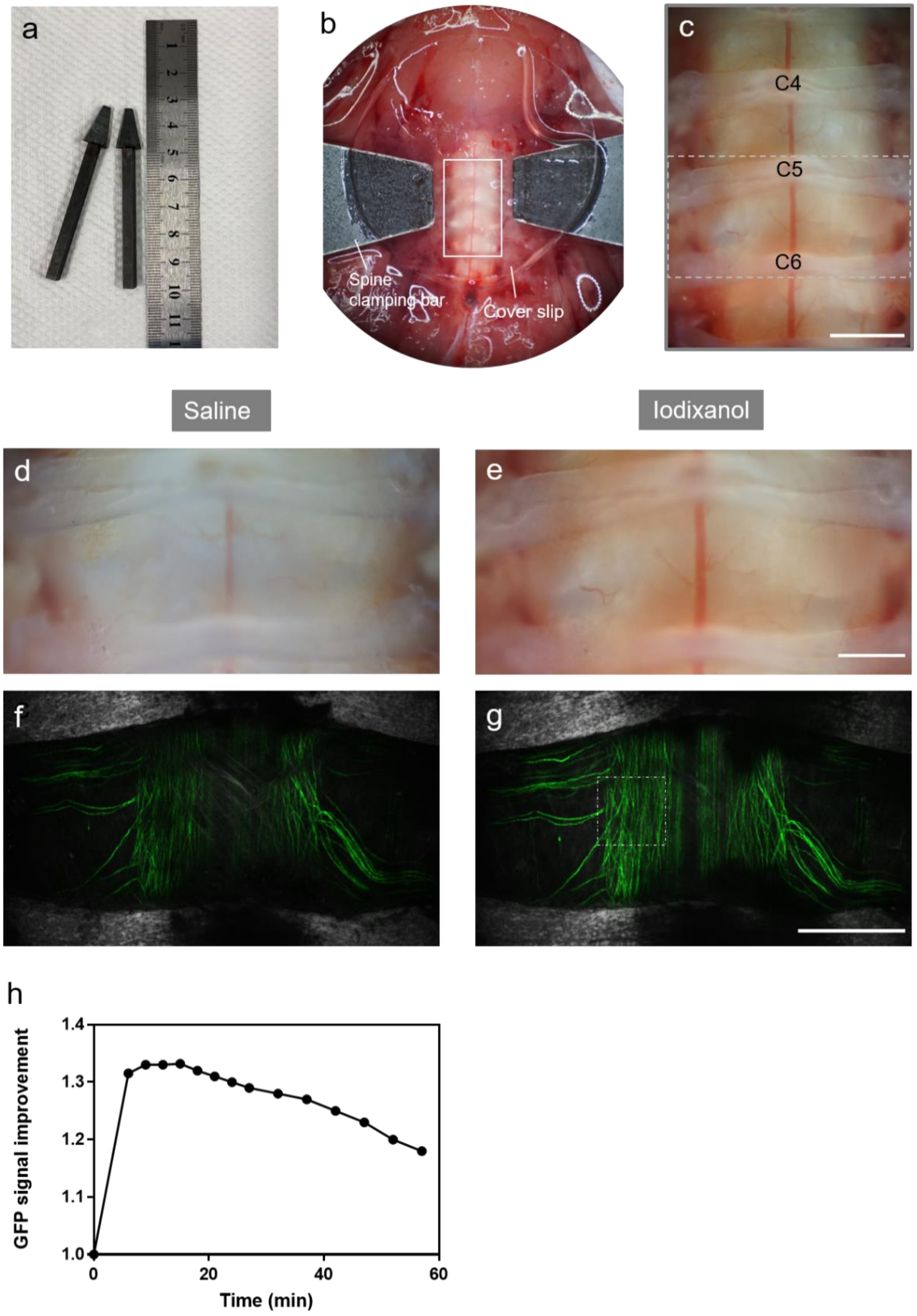

Supplementary Figure 9. ***In vivo*** **spinal cord imaging through intervertebral LF windows in the cervical region.** (a) Spine clamping bars for cervical spinal cord stabilization. (b) Bright-field image showing cervical spinal cord preparation scheme for *in vivo* imaging. (c) Magnified bright-field image in

the box region of (b) showing the optically cleared intervertebral LF window from C3 to C7. Scale bar, 1 mm. (d-g) Bright-field and TPEF images of the C5-C6 LF window in the dashed box region of (c) before (d, f) and after (e, g) optical clearing.. Green: YFP-labelled axons; Gray: SHG signal of collagen and other connective tissues. Scale bar, 500 μm. (h) GFP signal improvement as functions of time after a single application of Iodixanol. GFP intensity was calculated as the average of the brightest 0.1% pixels in the maximal projection images of axons in the dot-dashed box region in (g). Source data are provided as a Source Data file. Images shown in (d-g) are representative results of two experiments in two mice.

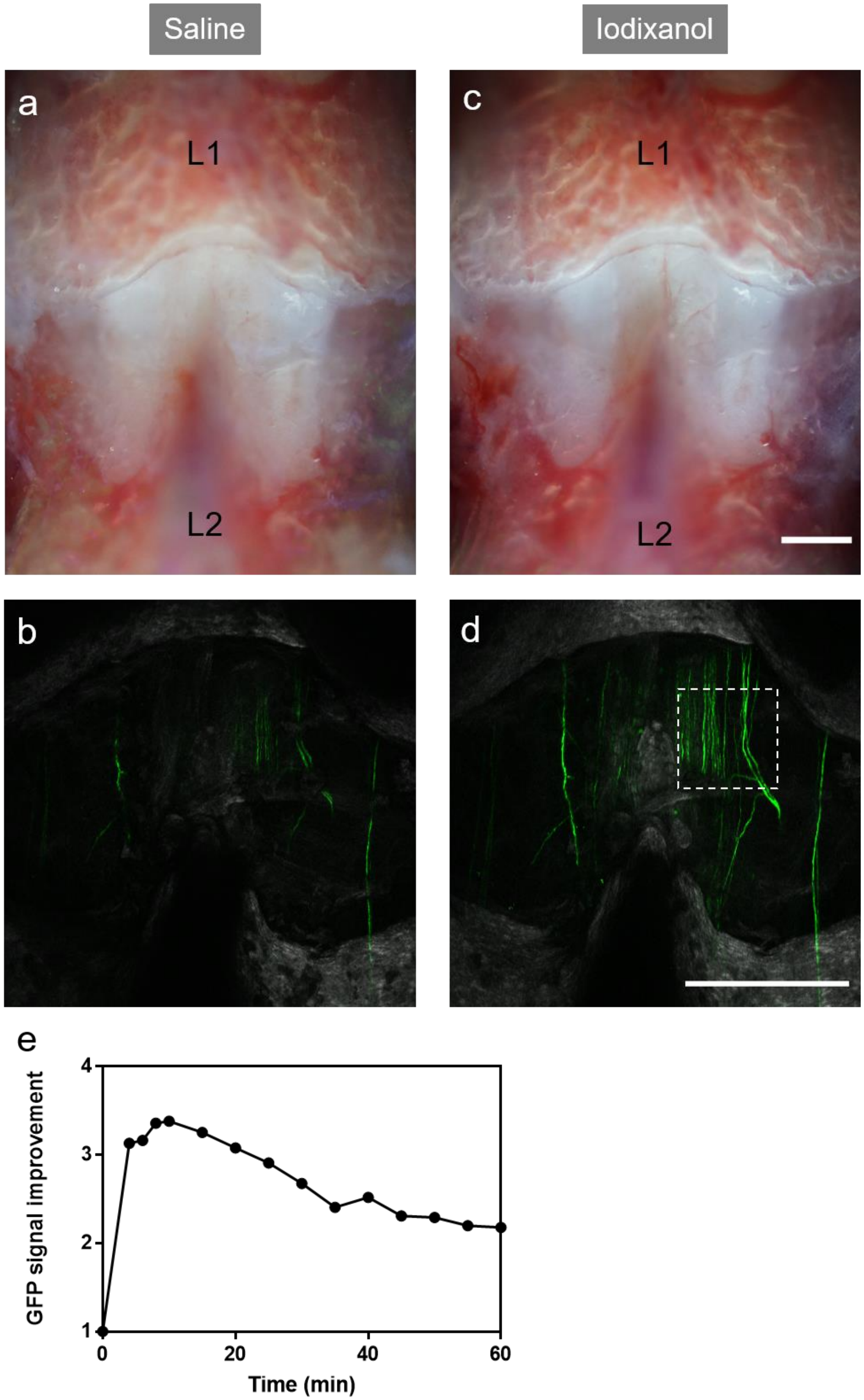

Saline
Iodixanol
a
L1
L2
c
L1
L2
b
d
e
GFP signal improvement
Time (min)
4
3
2
1
0
20
40
60

Supplementary Figure 10. *In vivo* spinal cord imaging through intervertebral LF windows in the lumbar region. (a-d) Bright-field and TPEF images of the spinal cord in the lumbar vertebral region through L1-L2 LF window before (a, c) and after (b, d) optical clearing. Green: YFP-labelled axons; Gray: SHG signal of collagen and other connective tissues. Scale bar, 500 μm. (e) GFP signal improvement as functions of time after a single application of Iodixanol. GFP intensity was calculated as the average of the brightest 0.1% pixels in the maximal projection images of axons in the dashed box region in (d). Source data are provided as a Source Data file. Mouse stabilization scheme at the lumbar region is the same as the T12-T13 lower thoracic region. Images shown in (a-d) are representative results of two experiments in two mice.

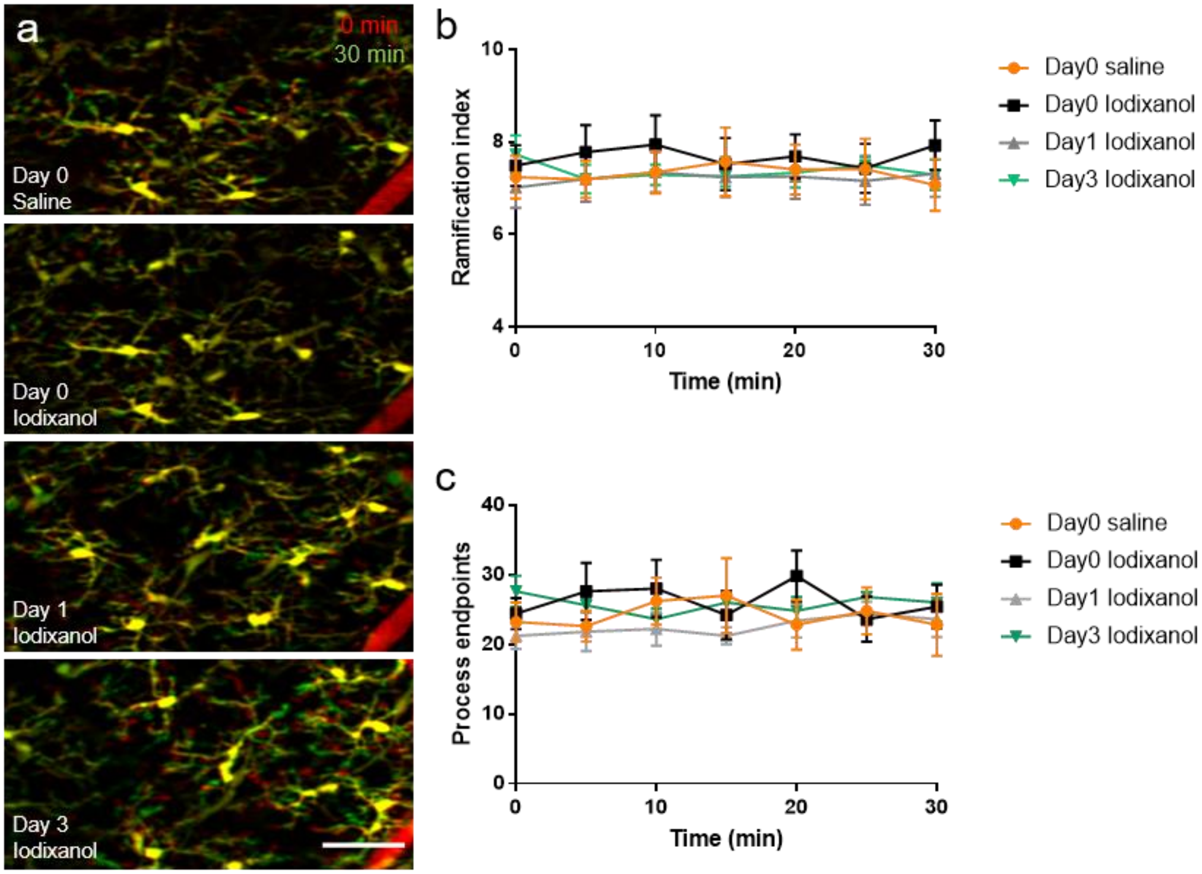

Supplementary Figure 11. Microglia do not show activation under Iodixanol administration. (a) Superimposed images of microglia at the same ROI at intervals of 30 min, showing process movement after saline/Iodixanol application at the indicated time. Blood vessels (red) labeled with Texas Red dextran are used as landmarks to navigate the same ROI. Scale bar, 50 μm. (b, c) Progress over time of the ramification index and process endpoints. n =5 microglial cells from the same ROI are selected for analysis. Error bar, s.e.m. Source data are provided as a Source Data file. Images shown in (a) are representative results of two experiments in two mice.

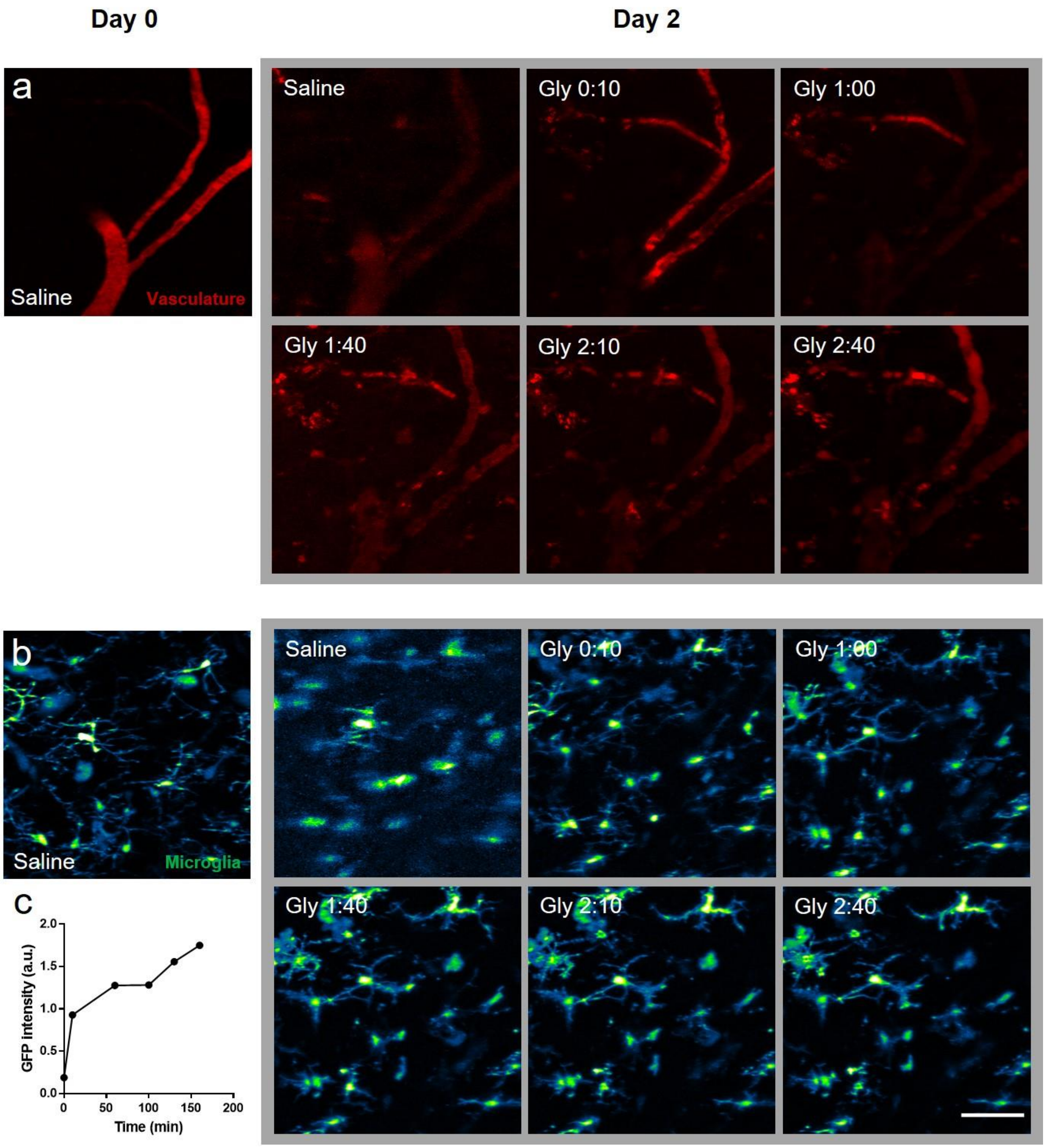

Supplementary Figure 12. Optical clearing of LF window with glycerol induced vasculature disruption and microglial activation. (a-b) Application of 70% v/v glycerol (G7757, Sigma-Aldrich) to the intervertebral window on day 2 helped to improve the image quality but induced vasculature disruption (a) and microglial activation (b), indicating glycerol toxicity to the spinal cord. Images of the same ROI taken on day 0 were used as a reference of normal microglial and vasculature morphology. The images

presented are the maximal projection of the acquired TPEF image stacks. Blood vessels are labeled with Texas Red dextran. Time is presented as hr:min. scale bar, 50 μm. (c) Microglia GFP intensity as functions of time after glycerol application. GFP intensity is the average intensity of the brightest 0.1% pixels in the microglia maximal projection images. Application of glycerol can help to improve the image contrast and resolution under the intervertebral window but with toxic effects that have been reported in previous studies[1]. A.u., arbitrary units. Source data are provided as a Source Data file.

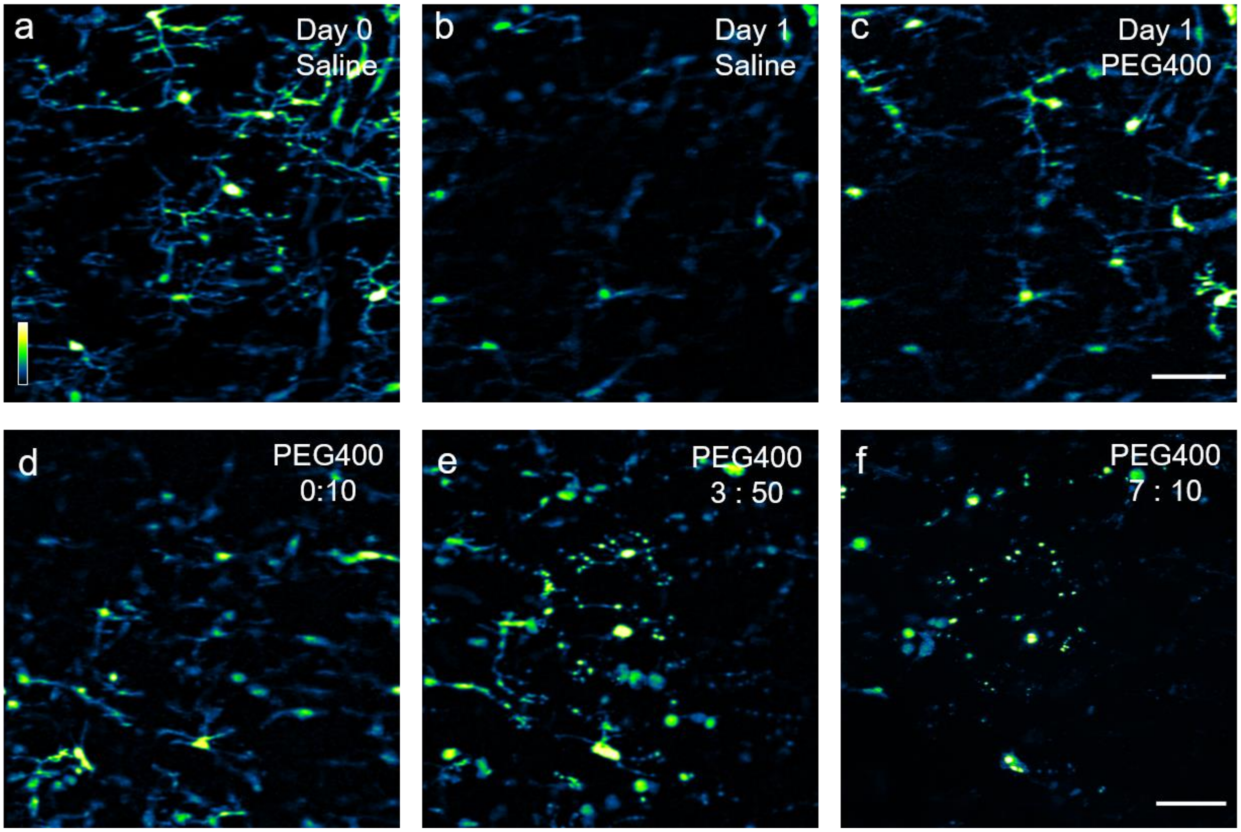

Supplementary Figure 13. Optical clearing of LF window with PEG400 has toxic effects. (a-c) Microglia maximal projection image on day 0 (a) and day 1 before (b) and 20 min after (c) application of 50% v/v PEG400 (P3265, Sigma-Aldrich). Image (b) and (c) are normalized to the same value. Scale bar, 50 μm. (d-e) Maximal projection image of microglia at the indicated time after PEG400 (50% v/v) application on day1. Enlarged soma size with beading processes of microglia was observed at the fourth hour after PEG400 application, which indicates the toxicity of PEG400 as an optical clearing agent for spinal cord imaging. Time is presented as hr:min. Scale bar, 50 μm.

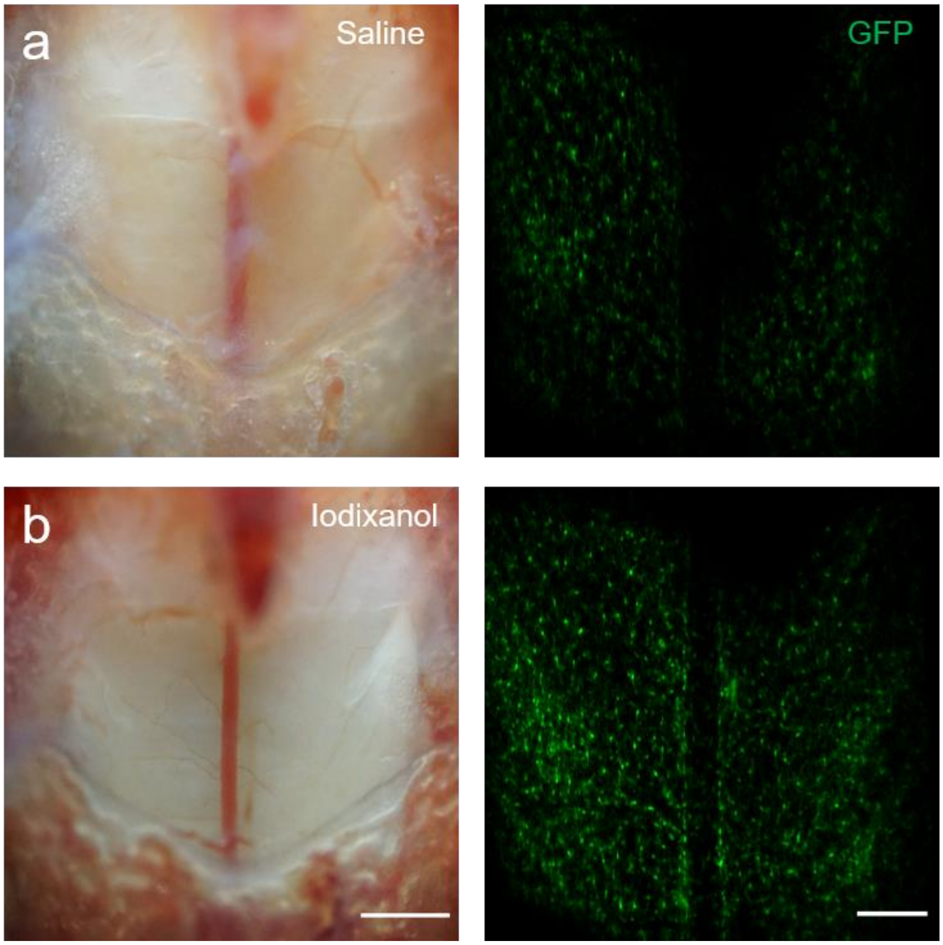

Supplementary Figure 14. The improvement of intervertebral window clarity by optical clearing on day 0. (a, b) Bright-field image and maximal projection of microglia TPEF image stack before (a) and after (b) optical clearing with 60% Iodixanol (w/v). Scale bar, 500 μm (bright-field), 200 μm (TPEF).

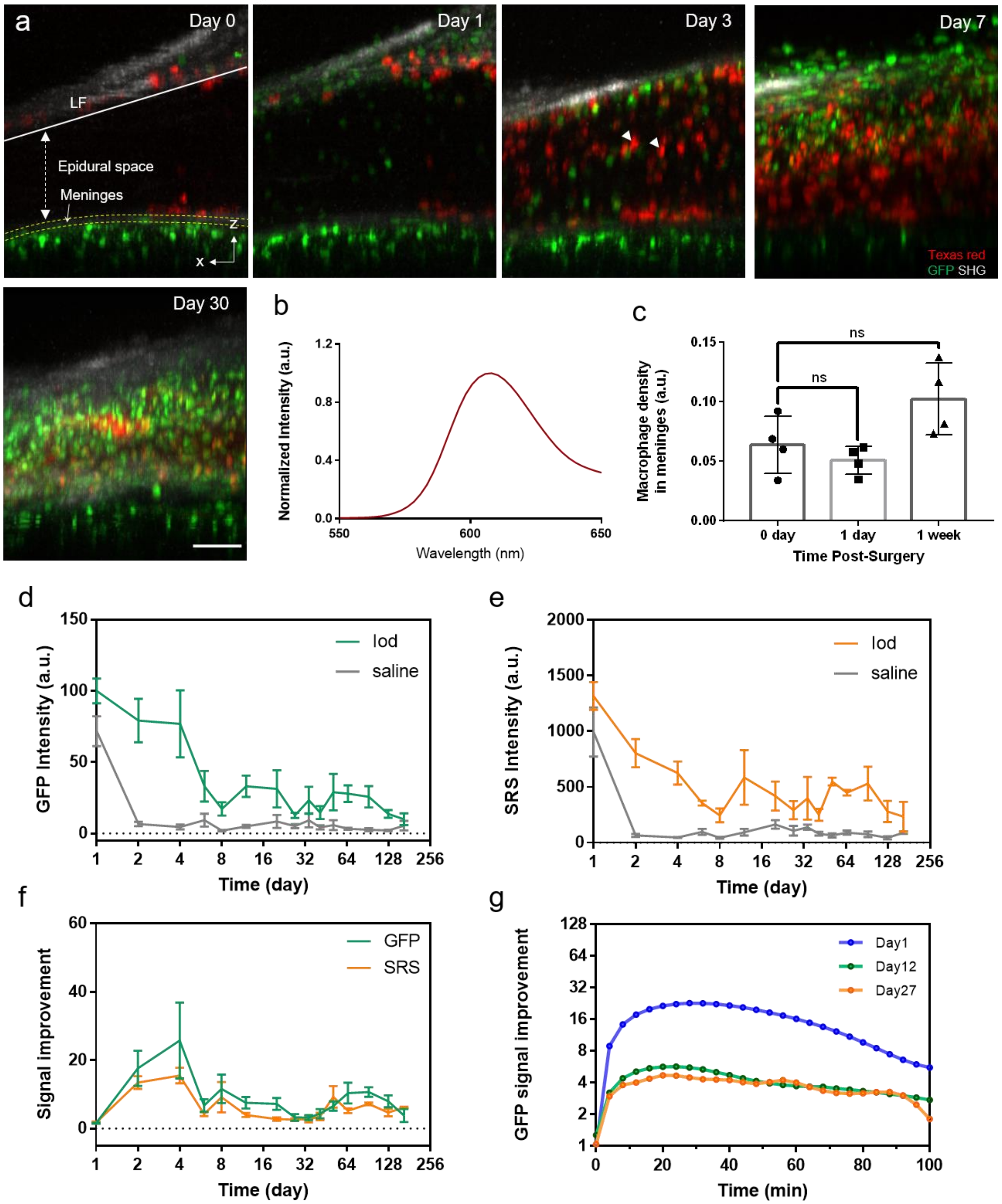

Supplementary Figure 15. The clearing effect of Iodixanol as a function of time (a) Maximal x-z projection images of the intervertebral window immersed in Iodixanol at the indicated time. Solid line shows the lower boundary of LF, yellow dashed area indicates the location of meninges and the double-

headed arrow indicates the area of epidural space. Red, blood vessels and invading, likely inflammatory cells in the epidural space labeled with Texas Red dextran; Green: GFP labeled microglia and other immune cells; Gray, SHG signals of connective tissues; Scale bar for x and z dimensions, 50 μm. (b) Spectral properties of the red fluorescence excited by 920 nm femtosecond laser, indicating the fluorescence of Texas Red. This strong red fluorescence was found in the perivascular space and layers above the spinal cord indicated by the white arrowhead in (a). (c) The macrophage density in the meninges at the same imaging area (300 $\mu m \times 300\ \mu m$) at indicated time points. n = 4 measurements from 4 mice for each time point. The meningeal layer was recognized by its SHG signal. One-way ANOVA: P = 0.6554 for 0 day vs. 1 day; P = 0.0772 for 0 day vs. 1 week. (d,e) GFP intensity of microglia (d) and SRS intensity of myelin (e) as functions of time after the first surgical procedure. At each time, GFP and SRS intensity were compared before and after Iodixanol application. (f) Improvement of GFP and SRS intensity by Iodixanol optical clearing as functions of time. At each time, the GFP and SRS intensity from the same ROI of each mouse were used for analysis. One to two ROIs were selected for analysis for each mouse, in total 4 mice were included for analysis. Error bar, s.e.m. (g) GFP signal improvement as functions of time after a single application of Iodixanol at the indicated time after the first surgery. GFP intensity is the average intensity of the brightest 0.1% pixels in the microglia maximal projection images. SRS intensity is the average intensity of the brightest 50% pixels in myelin maximal projection images. The improvement in signal is calculated as the ratio of signal intensity with and without optical clearing. A.u., arbitrary units. Source data are provided as a Source Data file.

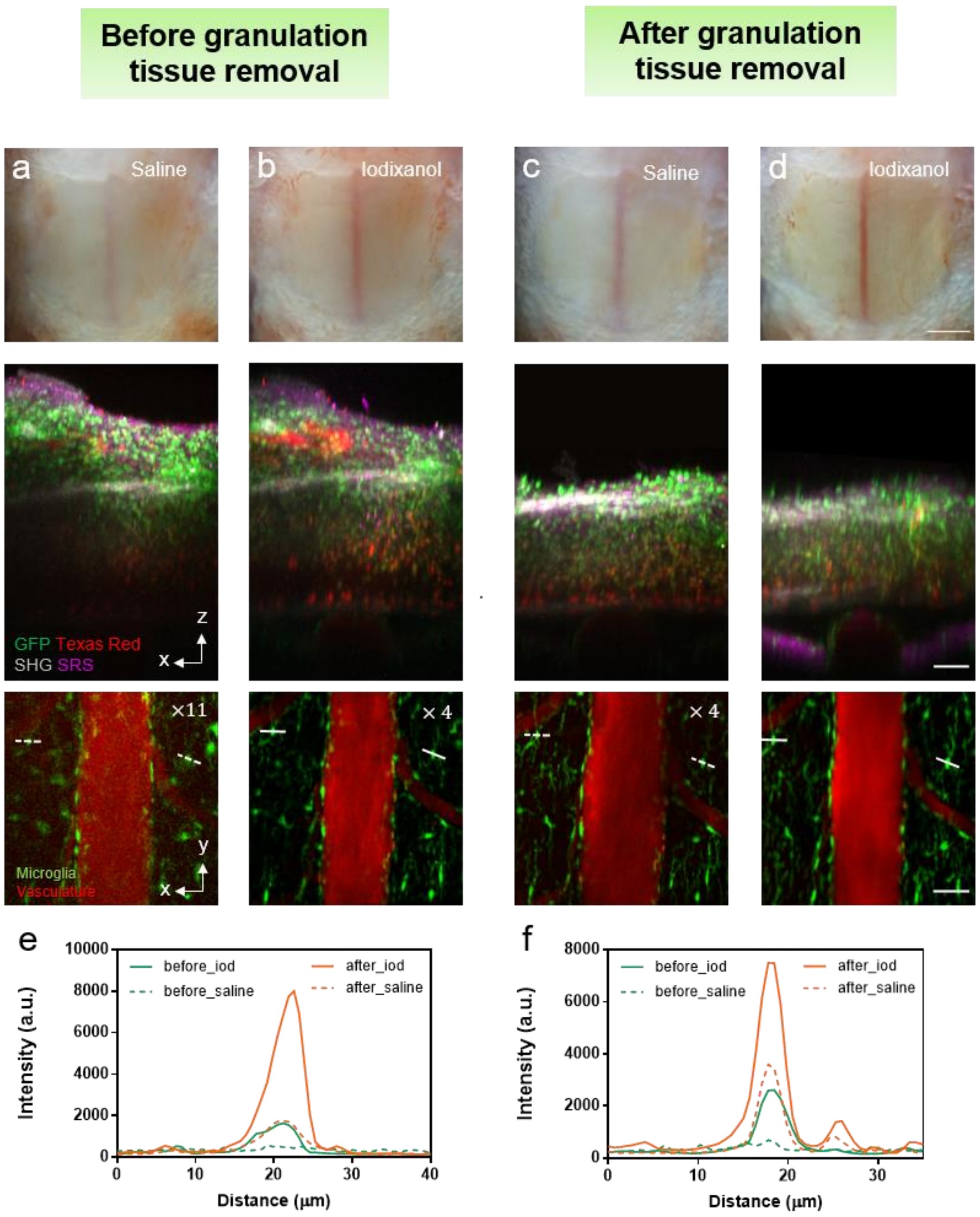

Supplementary Figure 16. Improvement of window clarity by granulation tissue removal. (a-d) Before and after removal of granulation tissue, comparison of the bright-field image, maximal x-z projection image of the intervertebral window and the corresponding x-y projection image of the spinal cord before and after Iodixanol application. x-y images of microglia and vasculature are normalized to the same value while images in (a-c) were enhanced digitally as indicated to improve visualization. All the images were taken at

the day 19 imaging session, 7 days post the last session (day 12). Red, blood vessels and immune-like cells labeled with Texas Red dextran; Green: GFP labeled microglia and other immune cells; Gray, SHG signals of connective tissues; Magenta, SRS signals of myelin and other tissues acquired at Raman shift of 2863.5 cm^{-1}. Scale bar, 500 μm for bright-field image; 50 μm for x-z multimodal and x-y fluorescence images, x, y, z dimensions are scaled in the same way. (e-f) Intensity profile along the solid and dashed line in (a-d). Green solid/dashed lines corresponds to the intensity profile of microglia with/without optical clearing before surgical removal of the granulation tissue, and yellow lines corresponds to the same intensity curves after tissue removal. A.u. arbitrary units. Source data are provided as a Source Data file.

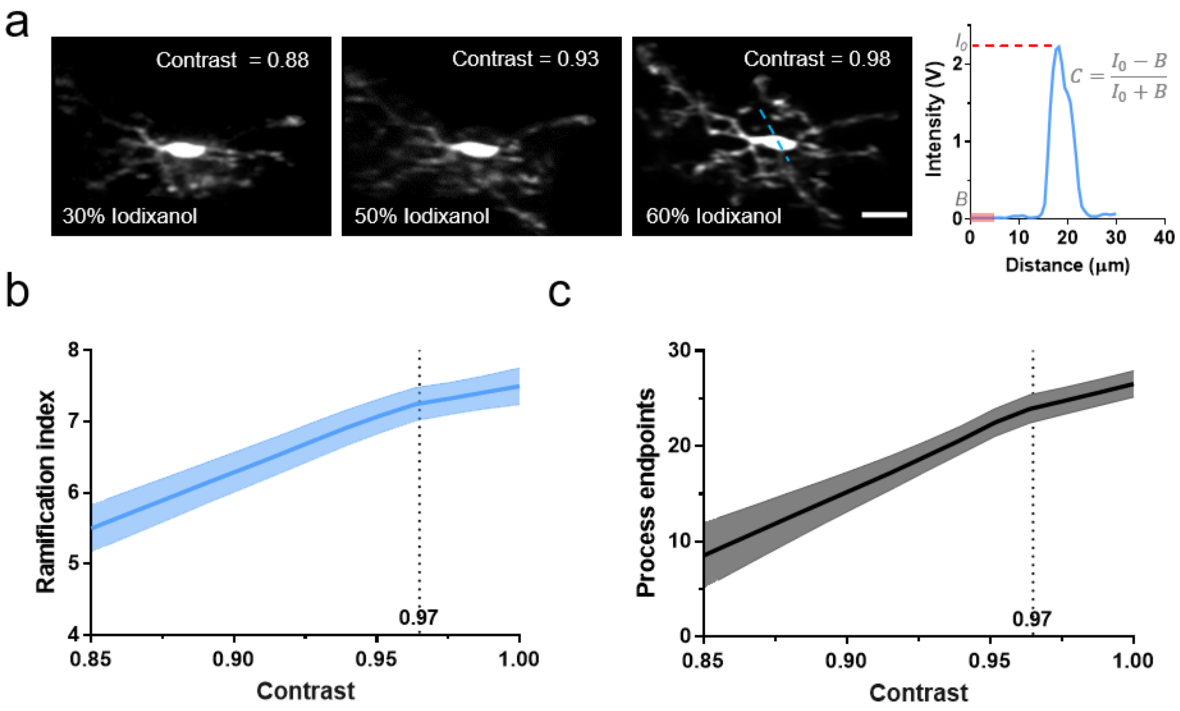

Supplementary Figure 17. Quantification of microglial morphology is dependent on the image contrast. (a) Images of the same microglia with different cell body contrast under different LF window clearance conditions. The definition of microglia cell body contrast is presented next to the intensity profile along the line shown in the image of 0.98 contrast (detailed definition of cell body contrast in the Methods section). Scale bar, 20 μm. (b, c) the dependence of the calculated ramification index and process endpoints on the contrast of microglial cells. The curves were acquired by averaging all single curves (n = 20) of each microglia. By curve fitting and slope analysis, image contrast of above 0.97 is used as the standard of microglial selection for morphological quantification. Error bar, s.e.m. Images shown in (a) are representative results of three experiments in three mice. Source data are provided as a Source Data file.

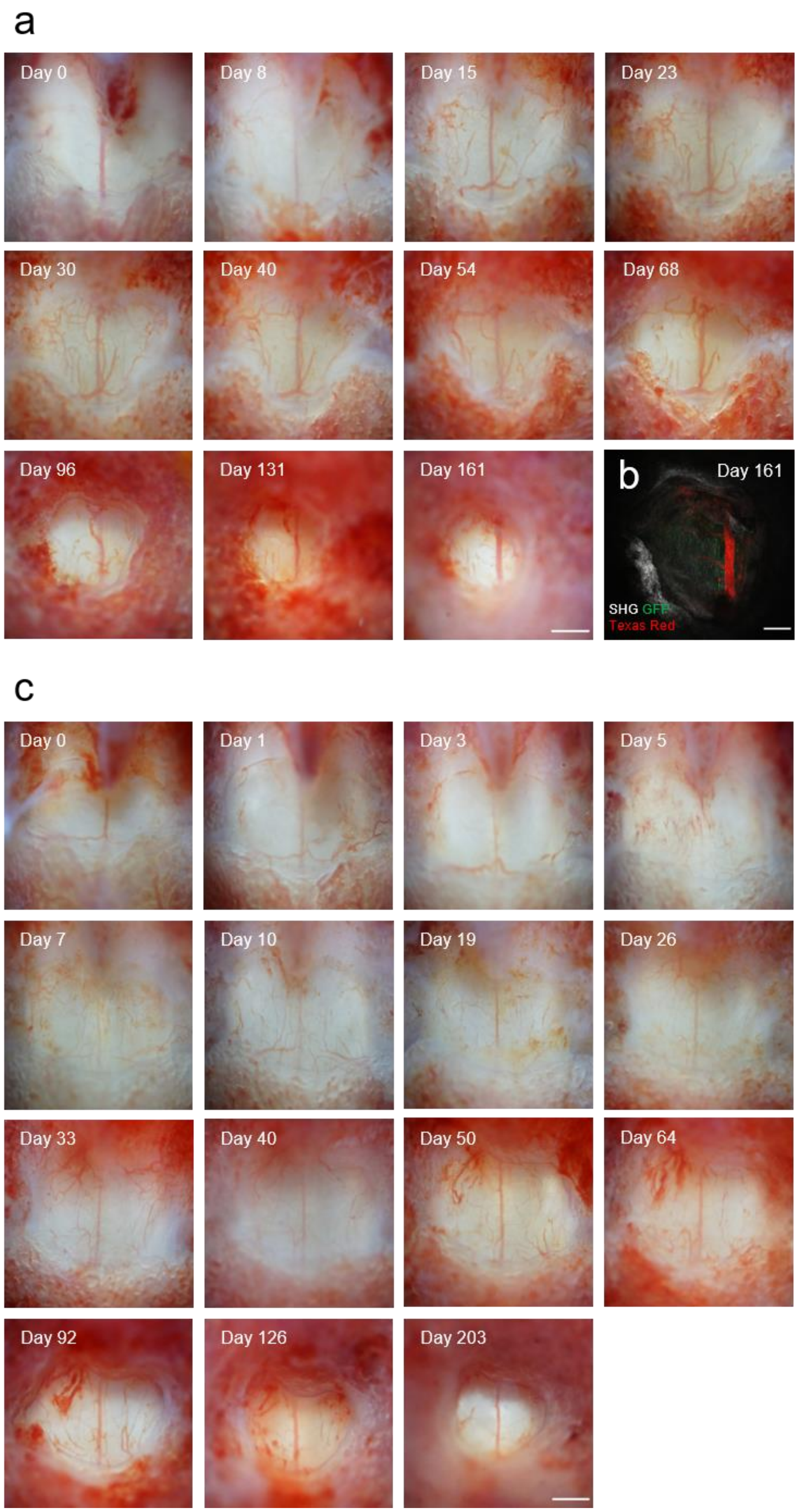
a
Day 0
Day 8
Day 15
Day 23
Day 30
Day 40
Day 54
Day 68
Day 96
Day 131
Day 161
b
Day 161
SHG GFP
Texas Red
c
Day 0
Day 1
Day 3
Day 5
Day 7
Day 10
Day 19
Day 26
Day 33
Day 40
Day 50
Day 64
Day 92
Day 126
Day 203

Supplementary Figure 18. Decrease of FOV of LF window with time. (a, c) Bright-field images of the intervertebral window from two mice at the indicated time after the first surgery, showing the decreased FOV after three months. Scale bar, 500 μm. (b) A two-photon image of the intervertebral window at day 161 shows strong SHG signal from the surrounding tissue which is difficult to remove, demonstrating the decrease of effective FOV. Red, blood vessels labeled with Texas Red dextran; Green: GFP labeled microglia; Gray, SHG signals of connective tissues; Scale bar, 200 μm.

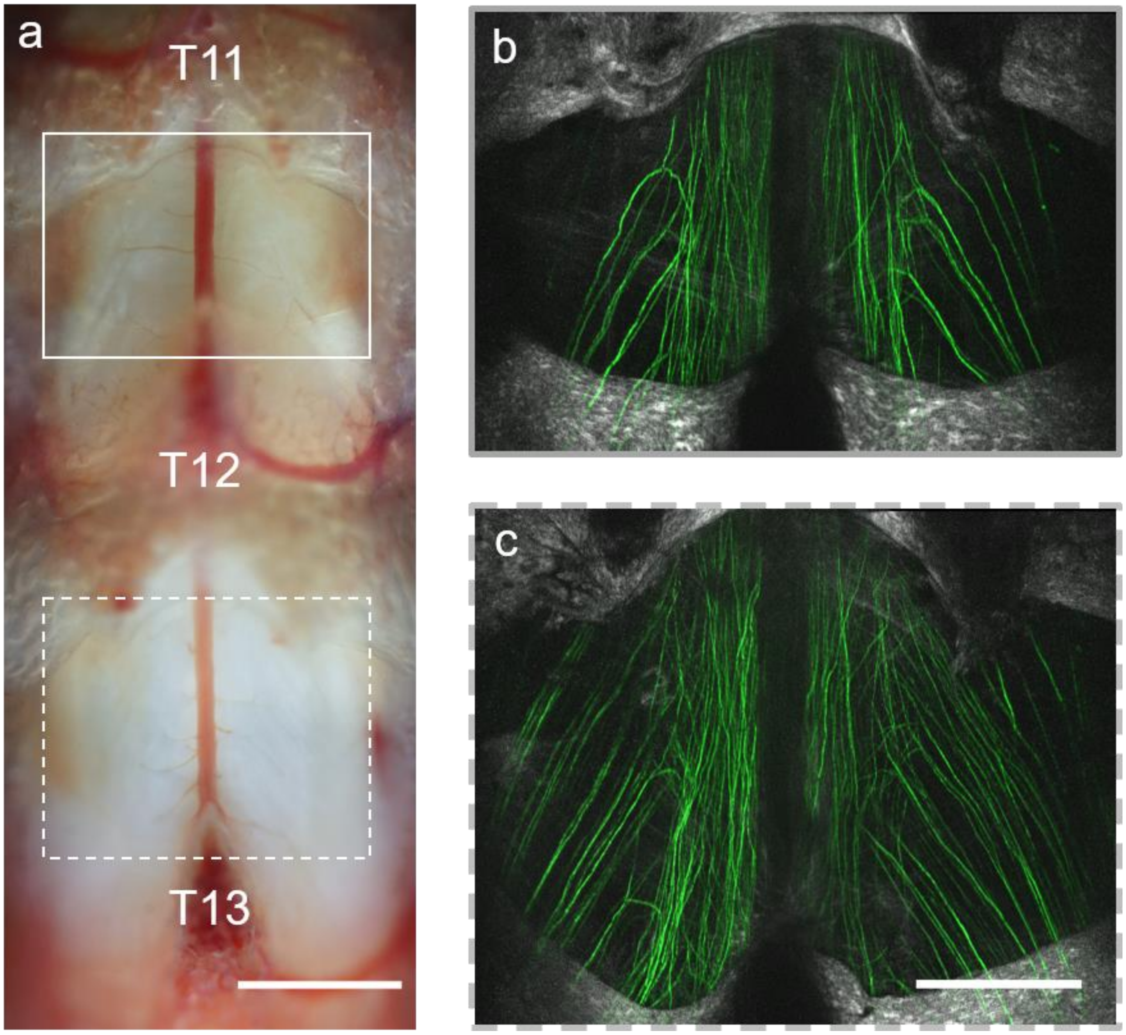

Supplementary Figure 19. Spinal cord imaging through two adjacent intervertebral LF at the lower thoracic region. (a) Bright-field image of the T11-T12 and T12-T13 intervertebral window on day 0. 60% Iodixanol (w/v) was administered for optical clearing. Scale bar, 1 mm. (b-c) Maximal two-photon projection images of the T11-T12 (b) and T12-T13 (c) LF window, corresponding to the solid and dashed box region in (a). Green: YFP labelled axons; Gray: SHG signal of collagen and other connective tissues. Scale bar, 500μm. Images shown in (a-c) are representative results of three experiments in three mice.

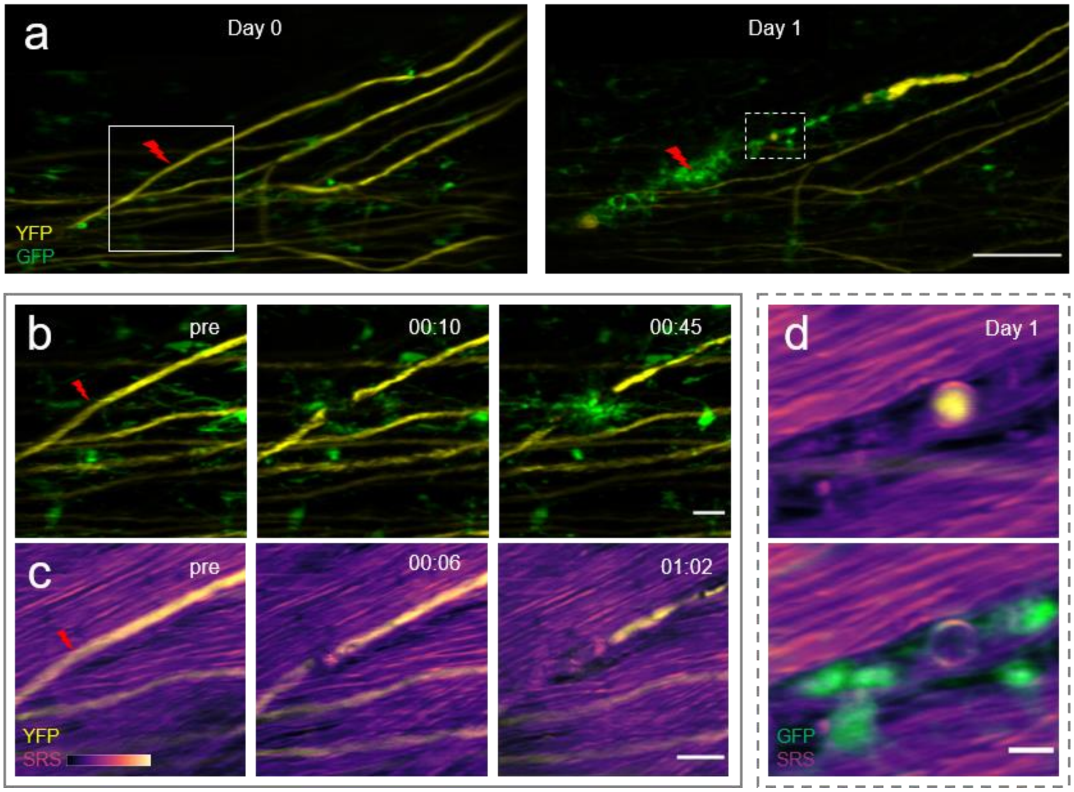

Supplementary Figure 20. Multimodal NLO imaging of axonal degeneration on day 0 and day 1 (a) Maximal projections of TPEF image stacks of axons (yellow) and microglia (green) at the indicated times. The lightning bolt symbol indicates the lesion site. Scale bar, 100 μm. (b,c) Maximal projection images of microglia (green) and axons (yellow) with its surrounding myelin in the solid box region in (a) before and after laser axotomy. Time is presented as hr:min. Scale bar, 20 μm. (d) Single myelin SRS image merged with axon (yellow) and microglia (green) TPEF image on day1 in the dashed box region in (a). Scale bar, 10 μm. SRS images of myelin were taken at Raman shift of 2863.5 cm^{-1}.

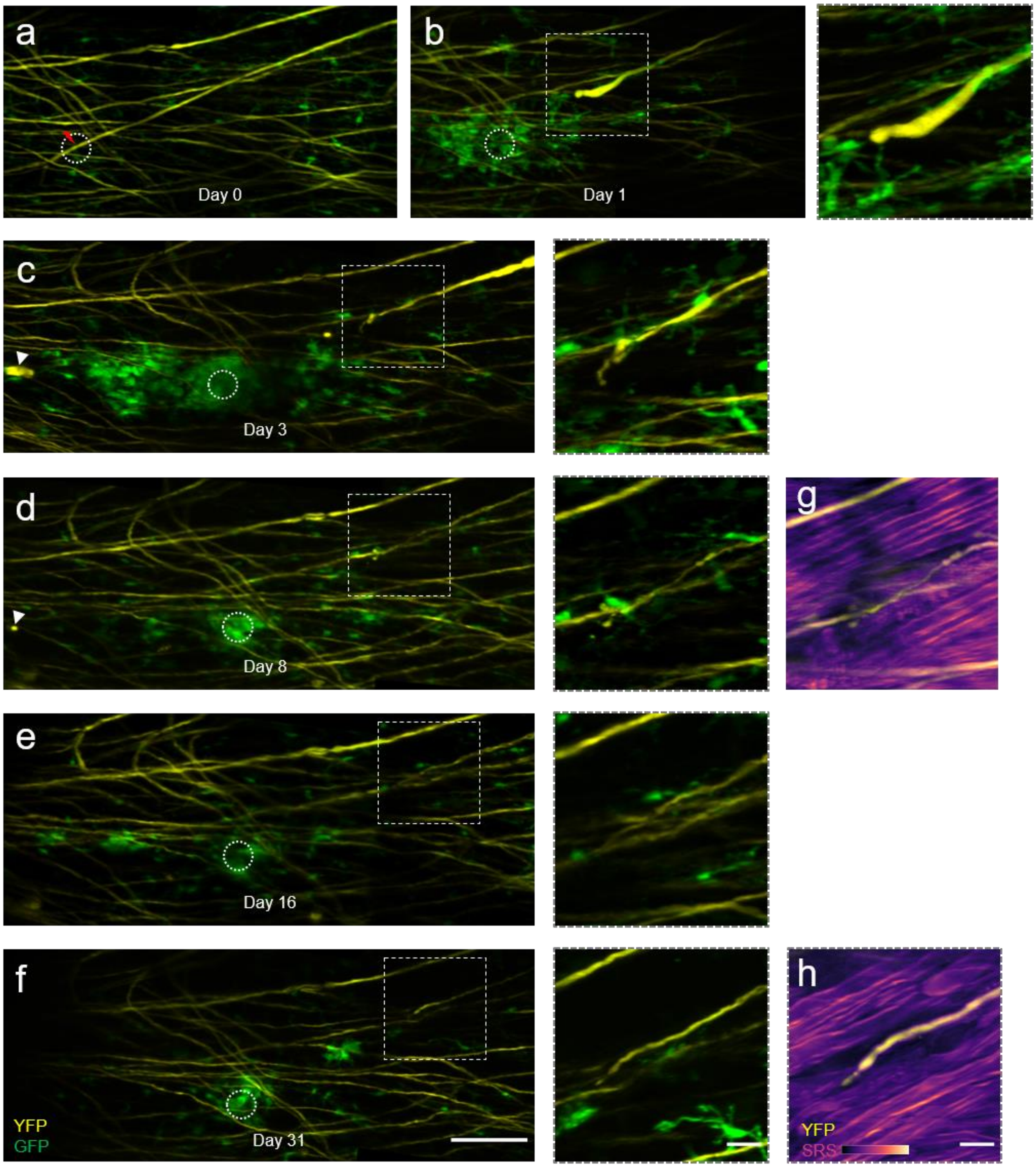

Supplementary Figure 21. Multimodal NLO imaging of axonal degeneration after laser axotomy with a large lesion. (a-f) Maximal projections of TPEF image stacks of axons (yellow) and microglia (green) at indicated times before and after laser axotomy. The lightning bolt symbol indicates the lesion site. The dashed circle indicates the size of injury. We determined the injury size based on observation of newly

generated fluorescence or myelin disruption. Magnified images (b-f) of the box region are shown on the right. Arrowheads in (c) and (d) indicate the location of axonal debris. Scale bar, 100 μm.(g, h) Single SRS image of myelin merged with axon TPEF image (yellow) at day 8 and day 31. SRS images of myelin were taken at Raman shift of 2863.5 cm^{-1}. Scale bar, 20 μm.

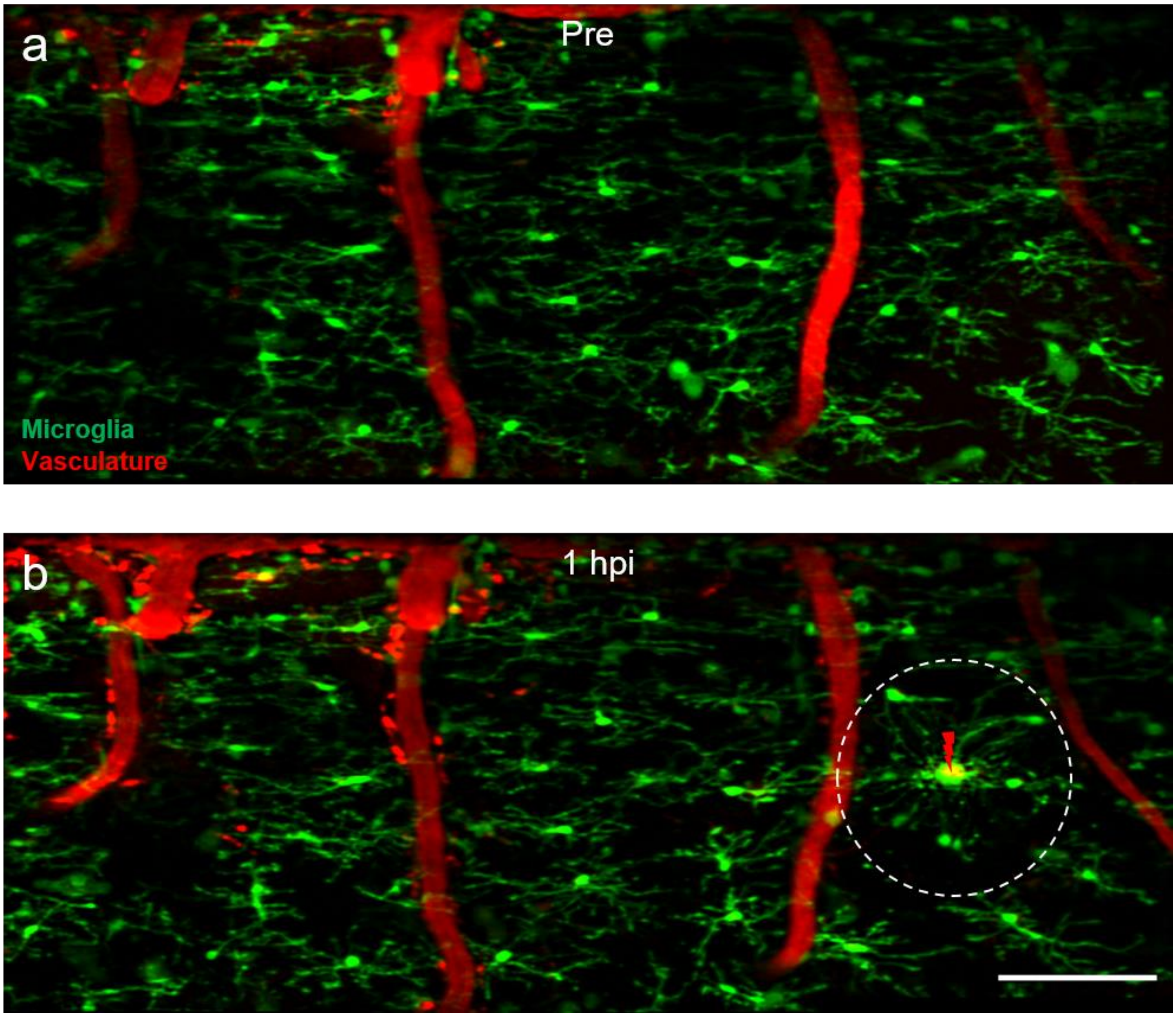

Supplementary Figure 22. Precisely confined laser injury only affects microglial behavior in a limited region. (a-b) Maximal projection image of microglia and vasculature before (a) and 1 hour after laser axotomy (b). The lightning bolt symbol indicates the lesion site. The dashed circle indicates the area in which the behavior of microglia is significantly affected by laser axotomy. The diameter of the dashed circle is 150 μm. Scale bar, 100 μm.

REFERENCES

1. Zhu, D. *et al.* Short-term and long-term effects of optical clearing agents on blood vessels in chick chorioallantoic membrane. *JBO* **13**, 021106 (2008).

Table S1 Accessories of the spinal cord stabilization device

Parts	Source	Material	Number	Remarks
Bottom plate with standing bars	Custom-made	Aluminium alloy	1	Standing bars and bottom plate should be manufactured separately
Holding plate	Custom-made	PMMA	1	To hold mouse body
Spine Clamping bar	Custom-made	Stainless steel	2	To secure mouse spine
Head bar	Custom-made	Stainless steel	2	To stabilize mouse head
M3 Knurled screw	Custom-made	Aluminium alloy	4	To fasten clamping bars and head bars
Adaptor rod	Custom-made	Aluminium alloy	2	
Post Clamp	Thorlabs (RA90TR/M)	N/A	2	To attach the holding plate to the standing bar and allow the holding plate moving along the standing bar by adjusting the screw of the post clamp;
M3 Screw	N/A	Aluminium alloy	2	To connect adaptor rod and holding plate
TACK IT	FABER-CASTELL	N/A		Attached to the head bar to better fit the shape of the mouse head and avoid applying strong mechanical pressure

Supplementary Note 1. Engineering drawings of the accessories of the spinal cord stabilization device

Supplementary Note 2. 3D view of spinal cord stabilization device.

Supplementary Note 1

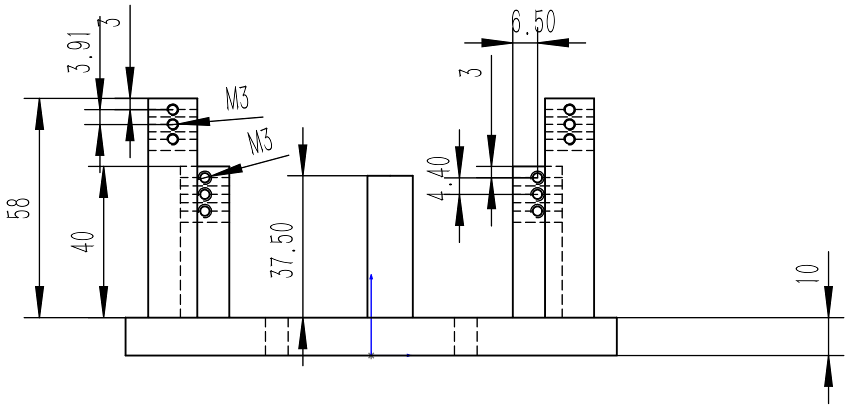

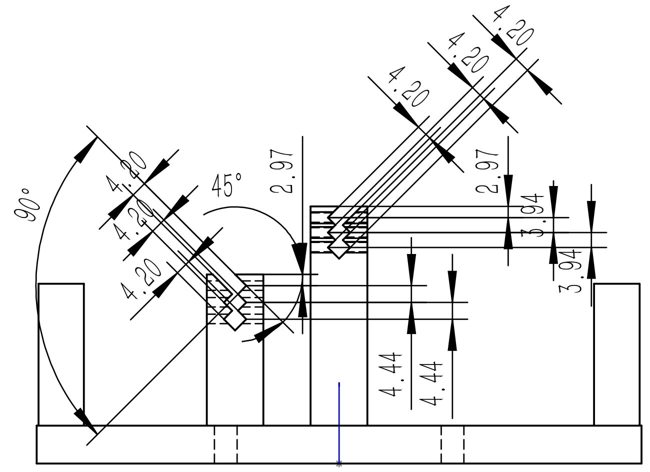

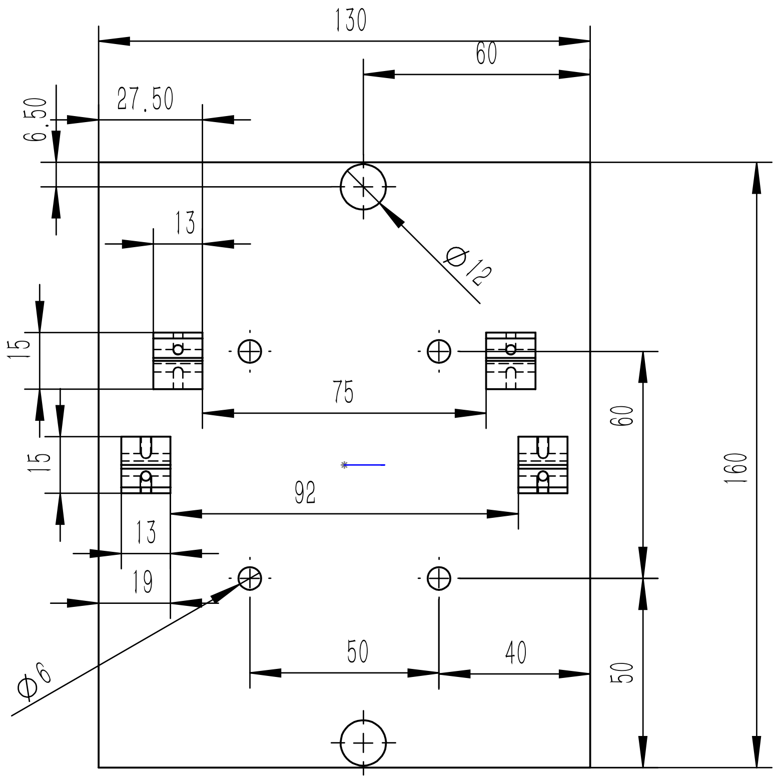

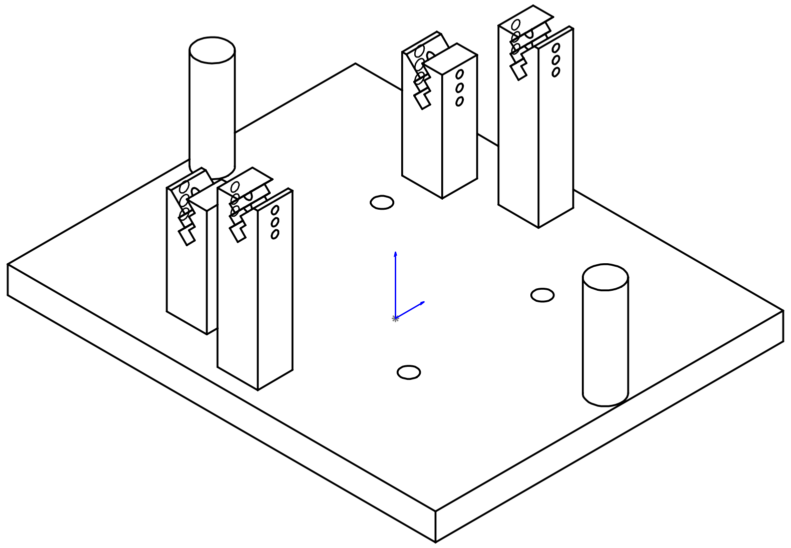

Bottom plate withstanding bars			
Aluminium alloy			unit: mm

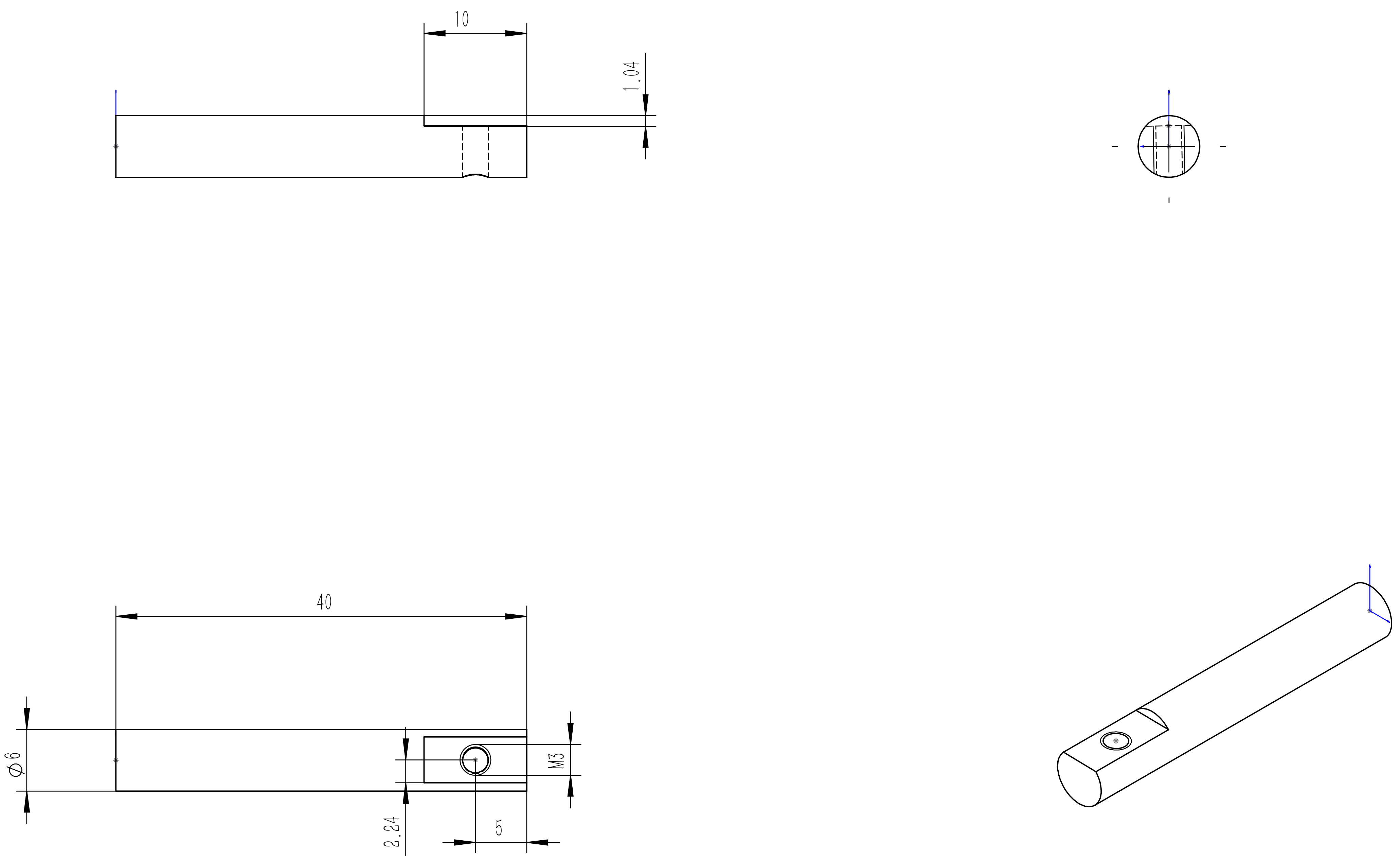

Adaptor rod			
Aluminium alloy			unit: mm
	1		

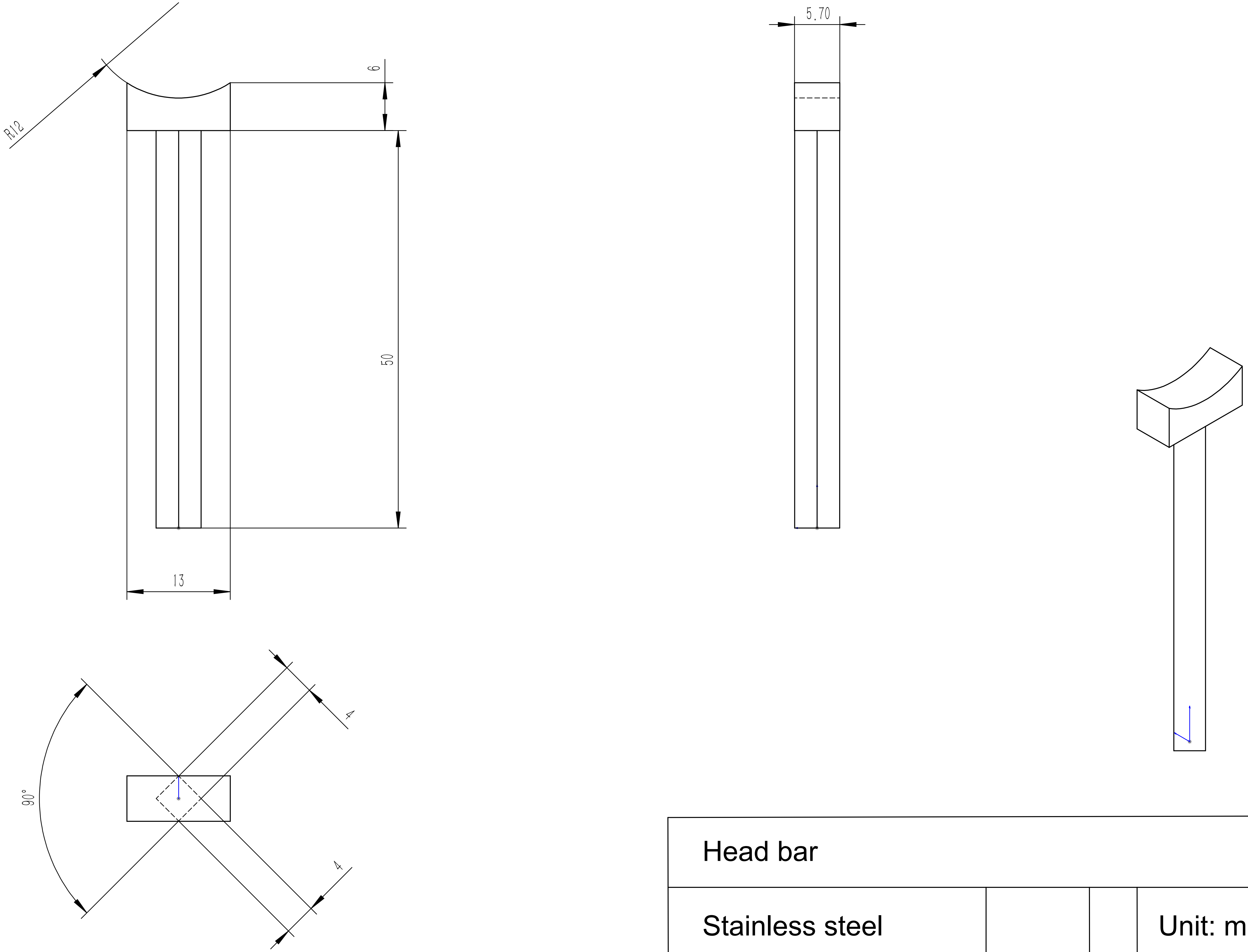

Head bar			
Stainless steel			Unit: mm

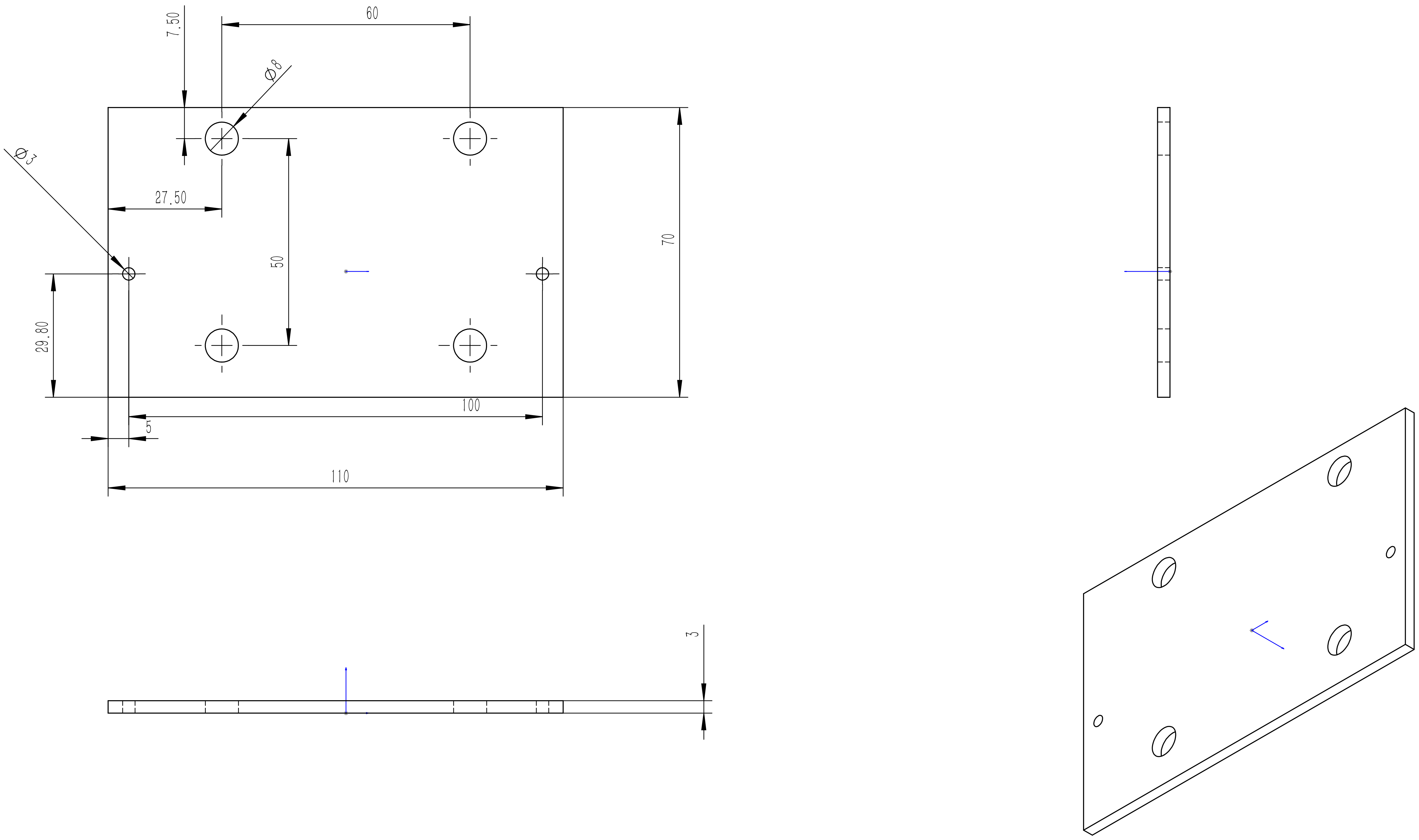

Holding plate			
PMMA			unit: mm

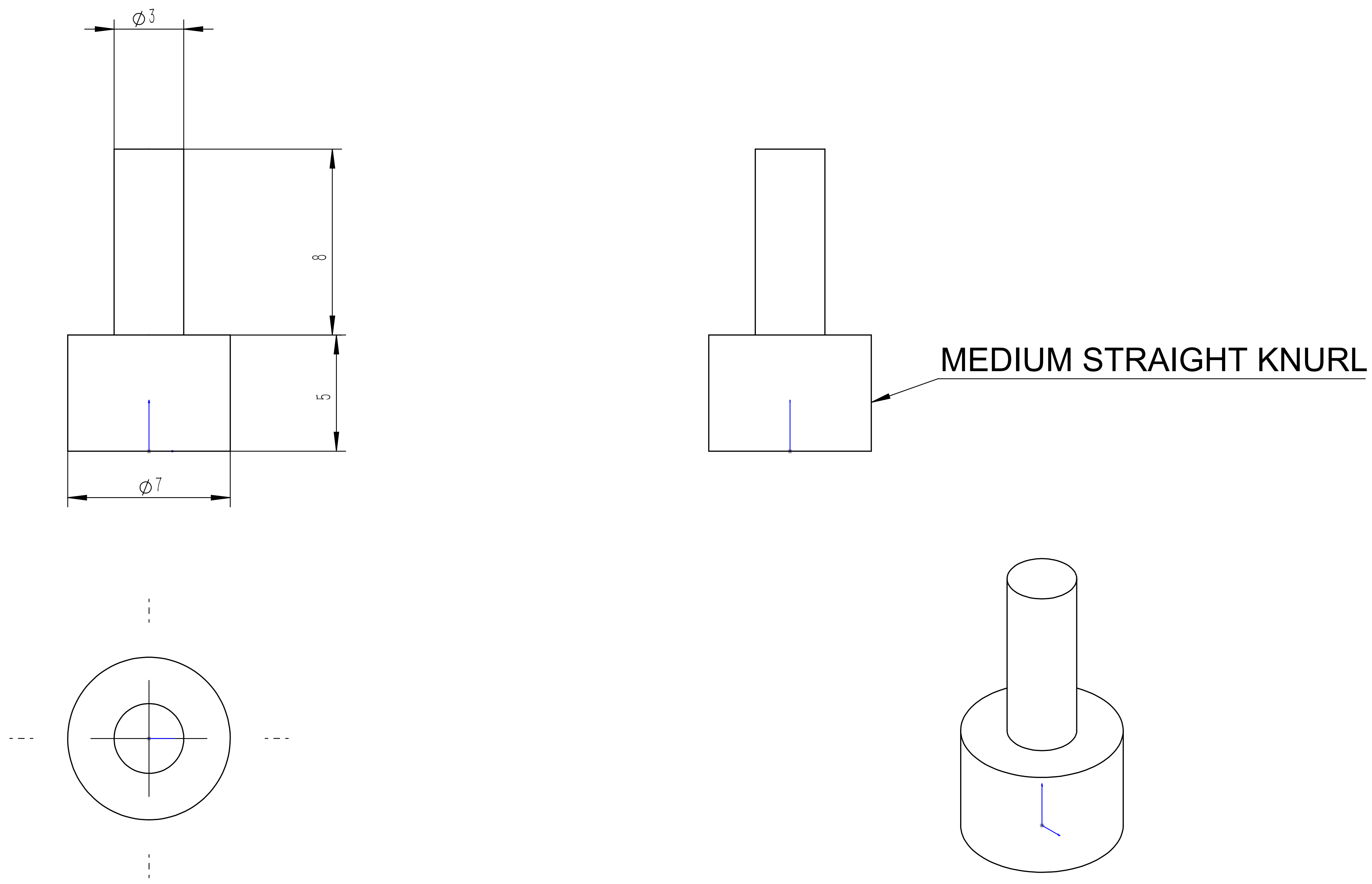

M3 Knurled screw			
Aluminium alloy			unit: mm

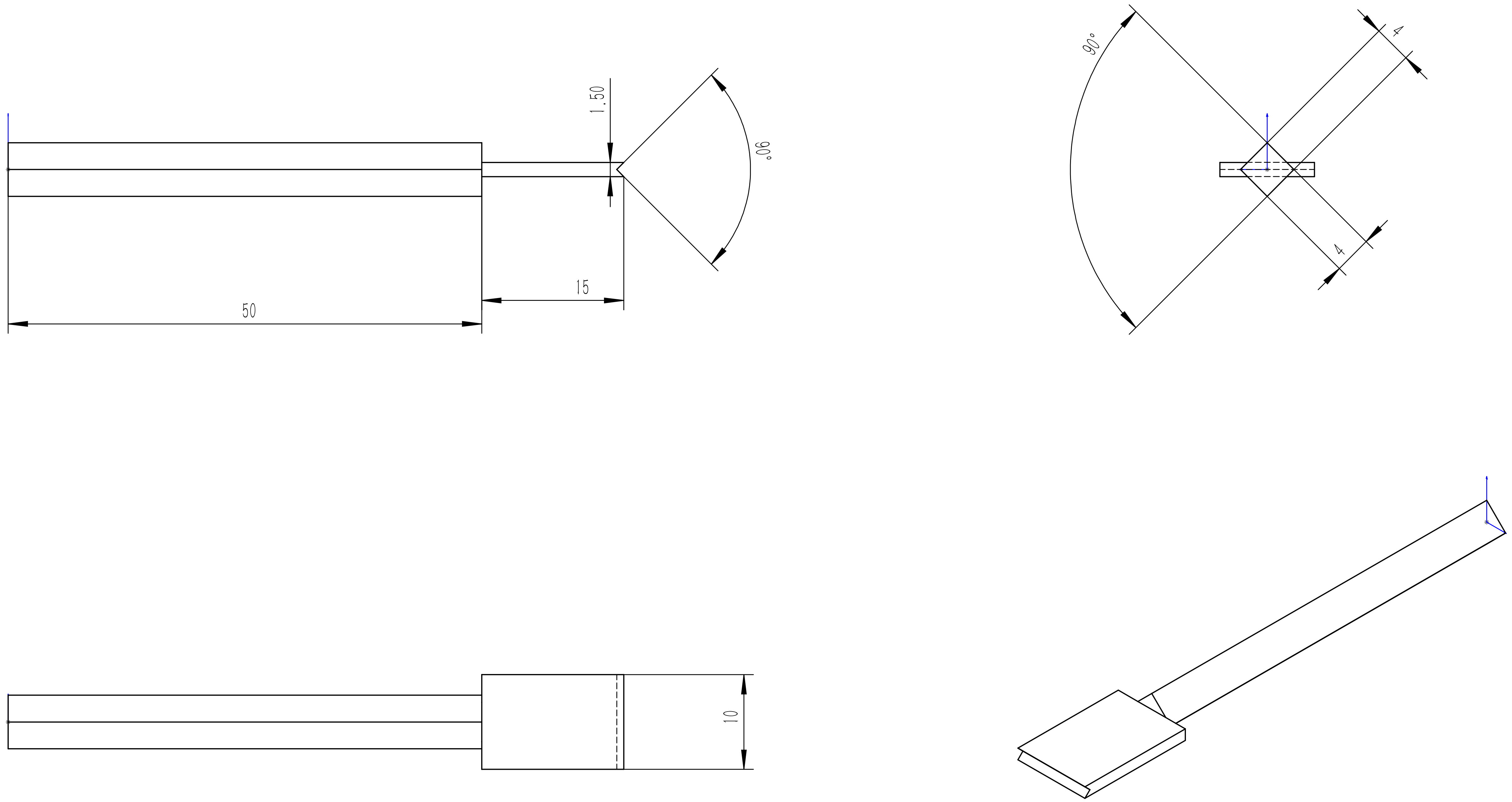

Spine clamping bar			
Stainless steel			unit: mm

Supplementary Note 2

Part Number:
Comments: Spinal Cord Stabilization Stage

Notes:

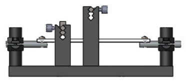
*Left

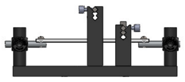
*Right

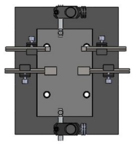
*Top

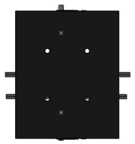
*Bottom

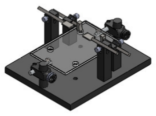
*Isometric

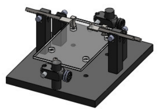
*Trimetric

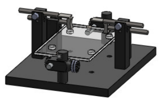
*Dimetric
