# Supplementary figures and images for: Long-term in vivo imaging of mouse spinal cord through an optically cleared intervertebral window
Source: Nat Commun. 2022 Apr 12;13:1959. doi: 10.1038/s41467-022-29496-x (PMC9005710; doi:10.1038/s41467-022-29496-x)

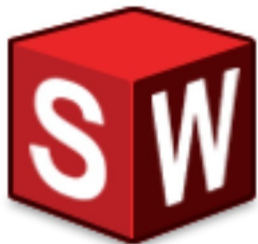

|  |
|--|
|  |
|--|

|  |
|--|
|  |
|--|

|  |
|--|
|  |
|--|

|  |
|--|
|  |
|--|

|  |
|--|
|  |
|--|

Supplement: Supplementary file 2 — Supplementary note 2 [file 41467_2022_29496_MOESM2_ESM.pdf]
