# Supplementary material for: Long-term in vivo imaging of mouse spinal cord through an optically cleared intervertebral window
Source: Nat Commun. 2022 Apr 12;13:1959. doi: 10.1038/s41467-022-29496-x (PMC9005710; doi:10.1038/s41467-022-29496-x)
Supplement: Supplementary file 4 — Reporting Summary [file 41467_2022_29496_MOESM4_ESM.pdf]

nature research

Corresponding author(s): Jianan Qu

Last updated by author(s): Mar 14, 2022

Reporting Summary

Nature Research wishes to improve the reproducibility of the work that we publish. This form provides structure for consistency and transparency in reporting. For further information on Nature Research policies, see our Editorial Policies and the Editorial Policy Checklist.

Statistics

For all statistical analyses, confirm that the following items are present in the figure legend, table legend, main text, or Methods section.

n/a	Confirmed	
☐	☒	The exact sample size (*n*) for each experimental group/condition, given as a discrete number and unit of measurement
☐	☒	A statement on whether measurements were taken from distinct samples or whether the same sample was measured repeatedly
☐	☒	The statistical test(s) used AND whether they are one- or two-sided<br>*Only common tests should be described solely by name; describe more complex techniques in the Methods section.*
☒	☐	A description of all covariates tested
☐	☒	A description of any assumptions or corrections, such as tests of normality and adjustment for multiple comparisons
☐	☒	A full description of the statistical parameters including central tendency (e.g. means) or other basic estimates (e.g. regression coefficient) AND variation (e.g. standard deviation) or associated estimates of uncertainty (e.g. confidence intervals)
☐	☒	For null hypothesis testing, the test statistic (e.g. *F*, *t*, *r*) with confidence intervals, effect sizes, degrees of freedom and *P* value noted<br>*Give P values as exact values whenever suitable.*
☒	☐	For Bayesian analysis, information on the choice of priors and Markov chain Monte Carlo settings
☒	☐	For hierarchical and complex designs, identification of the appropriate level for tests and full reporting of outcomes
☒	☐	Estimates of effect sizes (e.g. Cohen's *d*, Pearson's *r*), indicating how they were calculated

Our web collection on statistics for biologists contains articles on many of the points above.

Software and code

Policy information about availability of computer code

Data collection	Custom-written software (C#) was used for two-photon and stimulated Raman scattering imaging acquisition
Data analysis	ImageJ v1.53c (Fiji) and MATLAB 2019a were used for data analyses and image processing. GraphPad Prism 7 for figures and statistics. Imaris v7.4.2 was used for image 3D reconstruction. The MATLAB scripts for calculation of microglial ramification index and process endpoints are available online at “https://github.com/JaneWuhkust/Microglial-morphological-analysis”.

For manuscripts utilizing custom algorithms or software that are central to the research but not yet described in published literature, software must be made available to editors and reviewers. We strongly encourage code deposition in a community repository (e.g. GitHub). See the Nature Research guidelines for submitting code & software for further information.

Data

Policy information about availability of data

All manuscripts must include a data availability statement. This statement should provide the following information, where applicable:

- Accession codes, unique identifiers, or web links for publicly available datasets
- A list of figures that have associated raw data
- A description of any restrictions on data availability

All the data supporting the findings of this study are available within the paper and its supplementary information files. Other extended data figures are available from the corresponding author upon reasonable request. Owing to the size of the datasets, they are not available on a public data repository. Source data are provided with this paper.

Field-specific reporting

Please select the one below that is the best fit for your research. If you are not sure, read the appropriate sections before making your selection.

☒ Life sciences ☐ Behavioural & social sciences ☐ Ecological, evolutionary & environmental sciences

For a reference copy of the document with all sections, see nature.com/documents/nr-reporting-summary-flat.pdf

Life sciences study design

All studies must disclose on these points even when the disclosure is negative.

Sample size	No statistical methods were used to predetermine sample sizes, but our sample sizes were similar to those reported studies of chronic spinal cord imaging (Farrar, M. J. et al., Nature Methods 2012; Fenrich, K. K. et al., The Journal of Physiology 2012)
Data exclusions	Exclusion data was pre-established as the following: for preparation of intervertebral window, animals with spinal cord injured with bleeding during surgery were excluded from the rest in vivo imaging experiments. For preparation of intervertebral window with ligamentum flavum, animals with ligamentum flavum removed during surgery were excluded from the rest in vivo imaging experiments. Imaging sessions which were interrupted during the time course were terminated and that data were excluded from analysis due to the missing time-point.
Replication	Most of the experiments were replicated multiple times with the number of replication for both descriptive data and quantified results clearly indicated in the figure legend. All attempts at replication were successful.
Randomization	For all experiments, animals were randomly assigned to each groups.
Blinding	Investigators were not blinded to group allocation during all data analysis. For all data analysis involved in the manuscript, the quantified parameters and the data processing methods were strictly defined prior to quantification to avoid bias.

Reporting for specific materials, systems and methods

We require information from authors about some types of materials, experimental systems and methods used in many studies. Here, indicate whether each material, system or method listed is relevant to your study. If you are not sure if a list item applies to your research, read the appropriate section before selecting a response.

Materials & experimental systems

n/a	Involved in the study	
☒	☐	Antibodies
☒	☐	Eukaryotic cell lines
☒	☐	Palaeontology and archaeology
☐	☒	Animals and other organisms
☒	☐	Human research participants
☒	☐	Clinical data
☒	☐	Dual use research of concern

Methods

n/a	Involved in the study	
☒	☐	ChIP-seq
☒	☐	Flow cytometry
☒	☐	MRI-based neuroimaging

Animals and other organisms

Policy information about studies involving animals; ARRIVE guidelines recommended for reporting animal research

Laboratory animals	All strains of mice were on a C57BL/6J background and 2-6 months old. CX3CR1-GFP (JAX: 005582) heterozygous mice were used to visualize microglia for in vivo two-photon imaging and were bred to THY1-YFP-H (JAX:003782) mice for axon-glia interaction study. Both males and females were included in the study.
Wild animals	No wild animals were involved in this study.
Field-collected samples	No field-collected samples were involved in this study.
Ethics oversight	All animal procedures were conducted in accordance with the Guidelines of the Animal Care Facility of the Hong Kong University of Science and Technology (HKUST) and have been approved by the Animal Ethics Committee at HKUST.

Note that full information on the approval of the study protocol must also be provided in the manuscript.
